# Safety of HIF prolyl hydroxylase inhibitors for anemia in dialysis patients: a systematic review and network meta-analysis

**DOI:** 10.3389/fphar.2023.1163908

**Published:** 2023-05-24

**Authors:** Dinghua Chen, Yue Niu, Fei Liu, Yue Yang, Xue Wang, Ping Li, Xiangmei Chen

**Affiliations:** ^1^ Department of Nephrology, First Medical Center of Chinese PLA General Hospital, Beijing, China; ^2^ Department of Urology, Chinese Academy of Medical Sciences and Peking Union Medical College, Beijing, China

**Keywords:** hypoxia-inducible factor prolyl hydroxylase inhibitors, chronic kidney disease, anemia, renal dialysis, adverse event, erythropoiesis-stimulating agent

## Abstract

**Aim:** We performed a systematic review and network meta-analysis evaluating the safety and efficacy of hypoxia-inducible factor prolyl hydroxylase inhibitors (HIF-PHIs) among dialysis chronic kidney disease patients.

**Methods:** Safety was evaluated with any adverse events (AEs), serious adverse events (SAEs), and 12 common events. Efficacy was mainly analyzed with hemoglobin response. All reported results were summarized using mean difference and risk ratio (RR) with 95% confidence interval (CI). Publication bias was assessed through funnel plots.

**Results:** Twenty trials (19 studies) with 14,947 participants were included, comparing six HIF-PHIs with erythropoiesis-stimulating agents (ESAs). No significant differences were indicated in overall AEs and SAEs between each HIF-PHI and ESA. The occurrence of gastrointestinal disorder was higher in enarodustat and roxadustat than in ESAs (RR: 6.92, 95% CI: 1.52–31.40, *p* = 0.01; RR: 1.30, 95% CI: 1.04–1.61, *p* = 0.02). The occurrence of hypertension was lower in vadadustat than in ESAs (RR: 0.81, 95% CI: 0.69–0.96, *p* = 0.01). The occurrence of vascular-access complications was higher in roxadustat (RR: 1.15, 95% CI: 1.04–1.27, *p*<0.01) and lower in daprodustat (RR: 0.78, 95% CI: 0.66–0.92, *p*<0.01) than in ESAs. In the risk of the other nine events, including cardiovascular events, no significant differences were observed between HIF-PHIs and ESAs. For hemoglobin response, network meta-analysis showed that compared with ESAs, significant increases were shown in roxadustat (RR: 1.04, 95% CI: 1.01–1.07, *p*<0.01) and desidustat (RR: 1.22, 95% CI: 1.01–1.48, *p* = 0.04), whereas noticeable reductions were indicated in vadadustat (RR: 0.88, 95% CI: 0.82–0.94, *p*<0.01) and molidustat (RR: 0.83, 95% CI: 0.70–0.98, *p* = 0.02). There was no significant difference between daprodustat and ESAs (RR: 0.97, 95% CI: 0.89–1.06, *p* = 0.47).

**Conclusion:** Although HIF-PHIs did not show significant differences from ESAs in terms of overall AEs and SAEs, statistical differences in gastrointestinal disorder, hypertension, and vascular-access complications were observed between HIF-PHIs, which deserved to be noted in clinical decision making.

**Systematic review registration:** This study is registered with PROSPERO (registration number CRD42022312252)

## 1 Introduction

Anemia, a common complication of CKD related to the heightened risk of cardiovascular events, increased red blood cell transfusion and decreased health-related quality of life (Eckardt et al., 2021). Erythropoiesis-stimulating agents (ESAs) (particularly epoetin and darbepoetin) and iron supplements have become the mainstays of treatment, avoiding the risk of blood transfusions while ensuring optimal hemoglobin target levels (Zheng et al., 2020; Crugliano et al., 2021). Nevertheless, studies have shown that high-dose ESAs are relevant to increased risks of cardiovascular events and infection (Raichoudhury and Spinowitz, 2021), as well as hospitalizations and mortality (Culleton et al., 2006). The FDA has also revisited the prescribing information with black-box warnings for epoetin alfa and darbepoetin alfa; patients are at greater risk of adverse outcomes when the hemoglobin target value is >11 g/dL (Amge, Inc, 2018; Amge, Inc. 2019).

Hypoxia-inducible factor (HIF) prolyl hydroxylase inhibitor (PHI), an orally active small molecule, is a new drug type prepared for anemic CKD patients (Brigandi et al., 2016). To promote erythropoiesis in the kidney and liver, HIF-PHI can emulate the natural reaction to hypoxia (Hong et al., 2013; Koury and Haase, 2015; Chen et al., 2019) and, thus, stimulate endogenous erythropoiesis (EPO) and EPO receptor production and then promote the maturation of Hb-filled red blood cells (Tang et al., 2021). In addition, it can improve the utilization of iron by reducing hepcidin and increasing iron transport to the bone marrow to improve anemia (Del Balzo et al., 2020).

Six agents of HIF-PHIs have been reported, including roxadustat, daprodustat, vadadustat, molidustat, enarodustat, and desidustat. Some have been approved for treating CKD-related anemia in China, the EU, the United Kingdom, and Japan (Dhillon, 2019; Fishbane et al., 2022), as well as the United States. A meta-analysis of randomized controlled trials including 6,518 patients showed that roxadustat could effectively remedy anemia in dialysis-dependent (DD) CKD patients compared with ESAs, reduce cardiac failure, and increase the risk of hypotension, vomiting, and arteriovenous fistula thrombosis (Lei et al., 2022). Another meta-analysis of seven trials including 7,933 patients indicated that daprodustat might have a better impact on dialysis-dependent patients in optimizing iron metabolism despite being non-inferior in improving anemia in both DD and non-dialysis-dependent (NDD) patients (Fu et al., 2022). While a meta-analysis that included 14 studies indicated that DD patients using HIF-PHIs had a higher risk of serious adverse reactions compared to those using EPO (Wu et al., 2022), a new meta-analysis containing 23 studies showed a significant difference in the risk of cardiac and kidney-related AEs in NDD patients (Zheng et al., 2023), while there is still a lack of direct comparison between HIF-PHIs, which restricts the clinical application of these agents in DD patients. Therefore, to provide evidence for their safety in clinical application, we conducted a systematic review and network meta-analysis of RCTs comparing HIF-PHIs versus ESAs, to summarize their pairwise comparison and overall safety and efficacy.

## 2 Methods

### 2.1 Search strategy and selection criteria

Preferred Reporting Items for Systematic Reviews and Meta-Analyses guidelines (PRISMA) were followed for this network meta-analysis. A systematic search of databases, including PubMed, Embase, Web of Science, Ovid-EMBR, the Cochrane Library, and Chinese databases (CNKI, Wanfang, and CMJD), was set from inception to 31 August 2022. We used the following combined free-text and mesh terms: “Renal Insufficiency, Chronic,” and “hypoxia-inducible factor prolyl hydroxylase inhibitors.” The entire search strategy is illustrated in Supplementary Material S1. For additional relevant literature, ClinicalTrials.gov and references in selected research and reviews were searched.

### 2.2 Inclusion and exclusion criteria

Clinical studies were included that met the following criteria: 1) studies for RCTs only; 2) studies including adult patients diagnosed with renal anemia in DD CKD; 3) regardless of race, studies eligible for inclusion received HIF-PHI as the treatment group; 4) studies consisted of a control group treated with ESAs (epoetin alfa, darbepoetin alfa, etc.) in the same setting and for the same period; and 5) studies reported one or more relevant outcomes: change in AEs, SAEs, hemoglobin (Hb) response, ΔHb, hepcidin, transferrin saturation (TSAT), total iron binding capacity (TIBC), ferritin, and serum iron. Studies containing any of the following conditions were excluded: 1) studies published as reviews, conference abstracts, letters, case reports, editorials, and expert opinions; 2) studies involving healthy individuals or the same patient cohort included in evaluating another study; and 3) studies with less than 8 weeks of treatment.

### 2.3 Data extraction and quality assessment

Two researchers independently collected information from each trial as follows: author, publication year, treatments per group, sample size, baseline Hb levels, duration of treatment, mean age, sex, efficacy, and safety results [changes in AEs, SAEs, Hb (ΔHb), Hb response, Δhepcidin, ΔTSAT, ΔTIBC, Δferritin, and Δserum iron]. AEs and SAEs were the primary outcomes. The Cochrane tool will assess the risk of bias in clinical trials. Five domains were evaluated: randomization, deviations from intended interventions, missing data, outcome measurement, and selection of the reported result. Each domain was assigned a judgment of high risk of bias, low risk of bias, or some concerns. The Cochrane Handbook V.5.1.0, Chapter 8, was followed strictly to make the judgment for each domain.

### 2.4 Statistical analysis

The outcomes are summarized using mean difference (MD) and risk ratio (RR) with 95% confidence interval (CI). While our significant findings were derived from a frequentist network meta-analysis, a conventional meta-analysis was performed in advance to compare HIF-PHIs overall with ESAs briefly. The overall heterogeneity of effect size was tested. If there was significant between-study heterogeneity ( $I^{2}>50\%$ ) in the primary outcome, mean change in hemoglobin level from baseline, a random-effects model would be used, and a fixed-effects model would be used otherwise. In addition, Cochran’s Q-statistics was calculated under the assumption of design-by-treatment interaction random-effects models to assess the consistency of networks (Higgins et al., 2012; Jackson et al., 2012; Jackson et al., 2014). Funnel plots evaluated publication bias. Rankings of treatments were generated by estimating their surface under the cumulative ranking (SUCRA) scores, which is a metric to assess which treatment is likely to be the most efficacious (0: treatment is certain to be the worst; 1: treatment is certain to be the best) in the context of network meta-analyses (Salanti et al., 2011; Rücker and Schwarzer, 2015), while the closer to 1, the higher the possibility of an adverse event. The SUCRA score is calculated in the function using the following formula:

$${SUCRA}_{i}=\frac{\sum_{j=1}^{n-1}{cum}_{ij}}{n-1},$$

where *i* = 1, 2, … , *n* is the index of some treatment, *n* is the number of all competing treatments, *j* = 1, 2, . . ., n−1 is the rank of the best treatments, and *cum* represents the cumulative probability of treatment *i* being among the *j* best treatments. The influence of mean age, sex ratio, and duration of treatment was investigated through subgroup analysis using the Bayesian model. Finally, the network meta-analysis is repeated using the Bayesian model for sensitivity analysis (Higgins et al., 2009; Rover, 2020). All analyses were conducted with R 4.2.0 via the packages net-meta version 2.1–0 and gemtc version 1.0–1.

## 3 Results

### 3.1 Study selection

**FIGURE 1:**
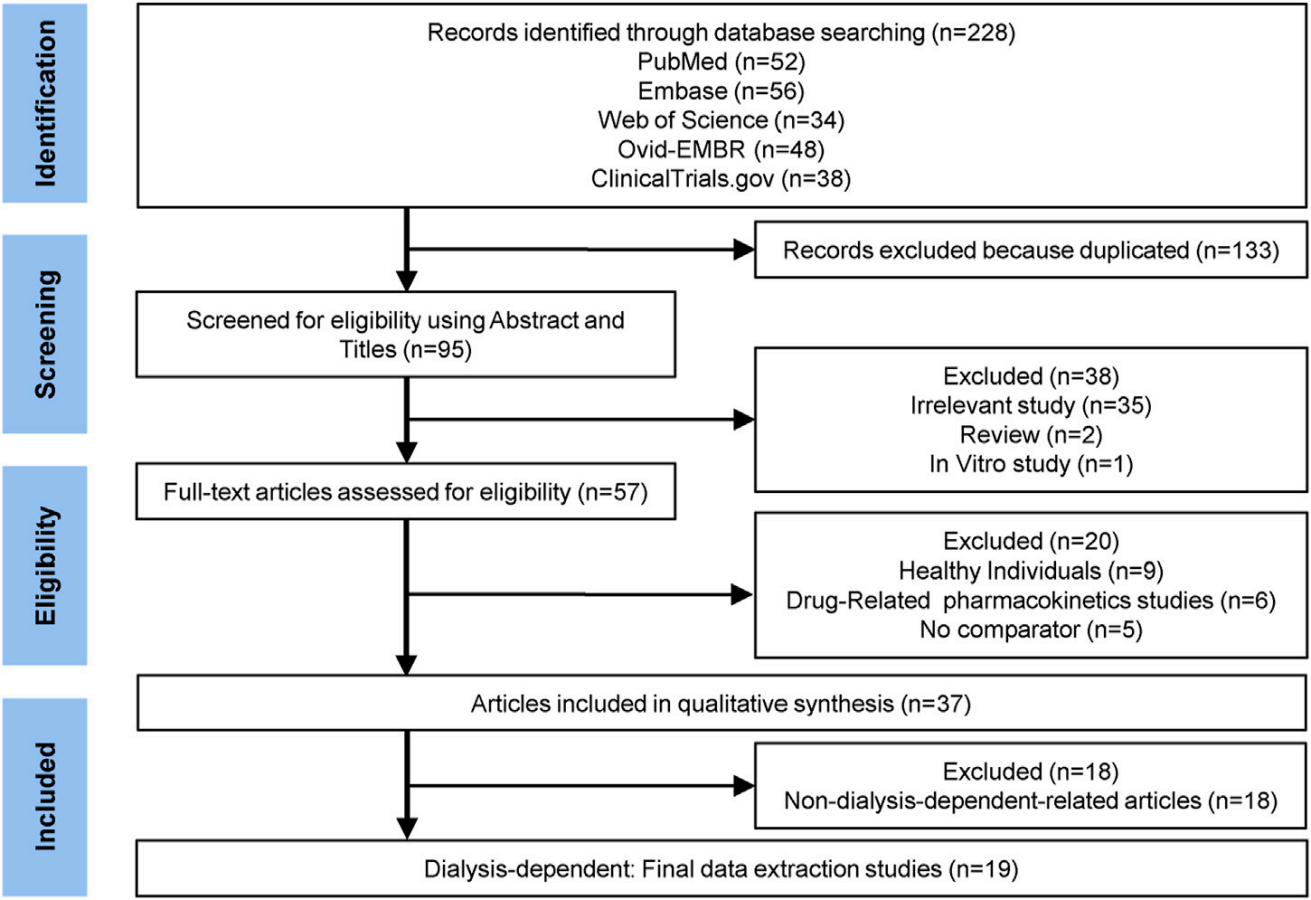
Flow chart of literature search and selection.

Initial literature searches are described in Figure 1. Ultimately, 19 DD-related eligible studies were included (Akizawa et al., 2019; Chen et al., 2019; Macdougall et al., 2019; Meadowcroft et al., 2019; Akizawa et al., 2020a; Akizawa et al., 2020b; Akizawa et al., 2021a; Akizawa et al., 2021b; Charytan et al., 2021; Csiky et al., 2021; Eckardt et al., 2021; Nangaku et al., 2021; Provenzano et al., 2021; Singh et al., 2021; Coyne et al., 2022; Fishbane et al., 2022; Gang et al., 2022; Hou et al., 2022; Singh et al., 2022). We searched the literature and finally included six agents of HIF-PHIs, including roxadustat (Chen et al., 2019; Akizawa et al., 2020a; Charytan et al., 2021; Csiky et al., 2021; Provenzano et al., 2021; Fishbane et al., 2022; Hou et al., 2022), daprodustat (Meadowcroft et al., 2019; Akizawa et al., 2020b; Singh et al., 2021; Coyne et al., 2022; Singh et al., 2022), vadadustat (Eckardt et al., 2021; Nangaku et al., 2021), molidustat (Akizawa et al., 2019; Macdougall et al., 2019; Akizawa et al., 2021b), enarodustat (Akizawa et al., 2021a), and desidustat (Gang et al., 2022). One of the studies described two different RCTs of vadadustat, and both trials compared vadadustat and darbepoetin, including incident DD CKD trial (369 participants) and prevalent DD CKD trial (3554 participants) (Eckardt et al., 2021).

### 3.2 Study characteristics and quality assessment

Characteristics of included research are depicted in Table 1. A total of 14,947 dialysis patients were included, with 7,787 participants in the HIF-PHI groups and 7,160 in the ESA groups. The network structures are shown in Supplementary Figure S1. The quality assessment of the included trials is displayed in Supplementary Figures S2–S9. Most trials were discerned to be of acceptable quality and judged to have a low risk of bias or some concerns, with the exception of one trial with an overall high risk of bias due to randomization, deviations from intended intervention, and missing data (Hou et al., 2022), evaluated by the Cochrane Collaboration’s risk of bias tool (Liu et al., 2020).

**TABLE 1:** Characteristics of the included comparisons in trials.

Author (year)	Comparison	Duration of treatment	Phase of study, location	Dosage of HIF-PHI	Baseline Hb(g/dL) (HIF-PHI/control)	Age (HIF-PHI/control)	HIF-PHI group(n) (male/female)	Control group (n) (male/female)
Chen et al. (2019)	Roxadustat vs. epoetin	26 weeks	III, China	Starting dose: 100 mg (45–60 kg) or 120 mg (≥60 kg) TIW	10.40 ± 0.70/10.50 ± 0.70	47.6 ± 11.7/51.0 ± 11.8	204 (126/78)	100 (58/42)
Akizawa et al. (2020a)	Roxadustat vs. darbepoetin	24 weeks	III, Japan	Starting dose: 70 or 100 mg TIW	11.02 ± 0.56/11.01 ± 0.60	64.6 ± 11.7/64.9 ± 10.1	150 (101/49)	151 (107/44)
Provenzano et al. (2021)	Roxadustat vs. epoetin	52 weeks	III, global	Starting dose: 70 mg (<70 kg) or 100 mg (≥70 kg) TIW	8.43 ± 1.04/8.46 ± 0.96	53.8 ± 14.7/54.3 ± 14.6	522 (309/213)	521 (307/214)
Hou et al. (2022)	Roxadustat vs. ESAs	24 weeks	III, China	Starting dose: 100 mg (45–60 kg) or 120 mg (≥60 kg) TIW	9.00 ± 1.40/9.00 ± 1.20	48.0 ± 12.0/48.3 ± 13.0	86 (47/39)	43 (25/18)
Csiky et al. (2021)	Roxadustat vs. daebepotin	52 weeks	III, global	Starting dose: 20, 50 and 100 mg TIW	10.75 ± 0.62/10.78 ± 0.62	61.0 ± 13.8/61.8 ± 13.4	414 (245/169)	420 (235/185)
Charytan et al. (2021)	Roxadustat vs. epoetin	52 weeks	III, US	Starting dose: 70–200 mg TIW based on the pre-study ESA dose	10.30 ± 0.66 10.31 ± 0.66	57.6 ± 13.6/58.4 ± 13.3	370 (187/183)	371 (215/156)
Fishbane et al. (2022)	Roxadustat vs. epoetin	208 weeks	III, global	Starting dose: ESAs naive:70 mg (45–70 kg) or 100 mg (70–160 kg) TIW ESAs users:70–200 mg TIW	10.10 ± 0.80/10.10 ± 0.90	53.5 ± 15.3/54.5 ± 15.0	1051 (625/426)	1055 (626/429)
Singh et al. (2021)	Daprodustat vs. epoetin	21 months	III, global	Starting dose: 4–12 mg q. d.	10.35 ± 0.97/10.39 ± 0.98	57.2 ± 14.3/57.3 ± 14.7	1487 (851/636)	1477 (847/630)
Meadowcroft et al. (2019)	Daprodustat vs. rhEPO, ESAs	24 weeks	Global	Starting dose: 4–12 mg q. d.	10.40 ± 0.66/10.60 ± 0.94	59.6 ± 13.3/59.7 ± 18.7	171 (108/63)	39 (26/13)
Akizawa et al. (2020b)	Daprodustat vs. darbepoetin	52 weeks	III, Japan	Starting dose: 4 mg q. d.	10.90 ± 0.80/10.80 ± 0.70	64.0 ± 10.0/64.0 ± 11.0	136 (91/45)	135 (89/46)
Singh et al. (2022)	Daprodustat vs. darbepoetin	52 weeks	III, global	Starting dose: 1–24 mg q. d.	9.50 ± 0.10/9.50 ± 0.10	53.7 ± 14.3/55.8 ± 15.7	157 (96/61)	155 (98/57)
Coyne et al. (2022)	Daprodustat vs. epoetin	52 weeks	III, global	Starting dose: 2–48 mg TIW	10.44 ± 0.83/10.59 ± 0.93	60 (50–69)/56 (46.5–65.5)	270 (149/121)	137 (81/56)
Eckardt et al. (2021)	Vadadustat vs Darbepoetin	52 weeks	III, global	Starting dose: 300 mg q. d. Maintenance dose: 150–600 mg q. d.	9.40 ± 1.10/9.20 ± 1.10	56.5 ± 14.8/55.6 ± 14.6	181 (107/74)	188 (113/75)
Eckardt et al. (2021)	Vadadustat vs. darbepoetin	52 weeks	III, global	Starting dose: 300 mg q. d. Maintenance dose: 150–600 mg q. d.	10.60 ± 0.90/10.20 ± 0.80	57.9 ± 13.9/58.4 ± 13.8	1777 (990/787)	1777 (1004/773)
Nangaku et al. (2021)	Vadadustat vs. darbepoetin	52 weeks	III, Japan	Starting dose: 300 mg q. d. Maintenance dose: 150–600 mg q. d.	10.73 ± 0.70/10.73 ± 0.70	66.0 ± 11.3/64.9 ± 11.7	162 (104/58)	161 (109/52)
Macdougall et al. (2019)	Molidustat vs. epoetin	16 weeks	IIb, US and Japan	Starting dose: 25–150 mg	10.50 ± 0.60/10.60 ± 0.50	59.0 ± 13.0/59.0 ± 9.0	157 (91/66)	42 (29/13)
Akizawa et al. (2019)	Molidustat vs. epoetin	52 weeks	IIb, Global	Starting dose: 15–150 mg q. d.	10.40 ± 0.70/10.50 ± 0.50	61.0 ± 12.0/59.0 ± 9.0	57 (33/24)	30 (23/7)
Akizawa et al. (2021)	Molidustat vs. darbepoetin	52 weeks	III, Japan	Starting dose: ESAs naive:75 mg q. d. user: 100/125/150 mg based on prior ESA dose	10.79 ± 0.65/10.87 ± 0.64	66.2 ± 10.3/64.8 ± 10.6	153 (91/62)	76 (49/27)
Akizawa et al. (2021)	Enarodustat vs. darbepoetin	24 weeks	III, Japan	Starting dose: 4 mg q. d.	10.79 ± 0.65/10.87 ± 0.70	63.2 ± 10.8/64.8 ± 10.3	86 (61/25)	86 (61/25)
Gang et al. (2022)	Desidustat vs. epoetin	24 weeks	III, India	Starting dose: 100 mg TIW	9.61 ± 0.99/9.55 ± 1.37	51.0 ± 14.0/51.0 ± 13.5	196 (135/61)	196 (134/62)

HIF-PHI: hypoxia-inducible factor prolyl hydroxylase inhibitor. ESA: erythropoiesis-stimulating agent. TIW: three times a week. q. d.: once daily.

### 3.3 Conventional meta-analysis

The safety was compared between HIF-PHIs overall and ESAs through conventional meta-analyses regarding AEs and SAEs. The differences were not significant in either AEs (RR: 1.00, 95% CI: 0.99–1.02, *p* = 0.72, I^2^ = 20.5%) or SAEs (RR: 0.99, 95% CI: 0.95–1.04, *p* = 0.76, I^2^ = 22.4%). The efficacy was compared between HIF-PHIs overall and ESAs regarding Hb response and ΔHb. Also, no statistical differences were indicated in the Hb response (RR: 1.01, 95% CI: 0.94–1.07, *p* = 0.83, I^2^ = 71.9%) and ΔHb (MD:0.05 g/dL, 95% CI: 0.04–0.15, *p* = 0.23, I^2^ = 85.4%). This result indicated that the individual effect of each HIF-PHI agent must be studied carefully, which led to our subsequent network meta-analysis.

### 3.4 Safety about treatment-emergent adverse events

**FIGURE 2:**
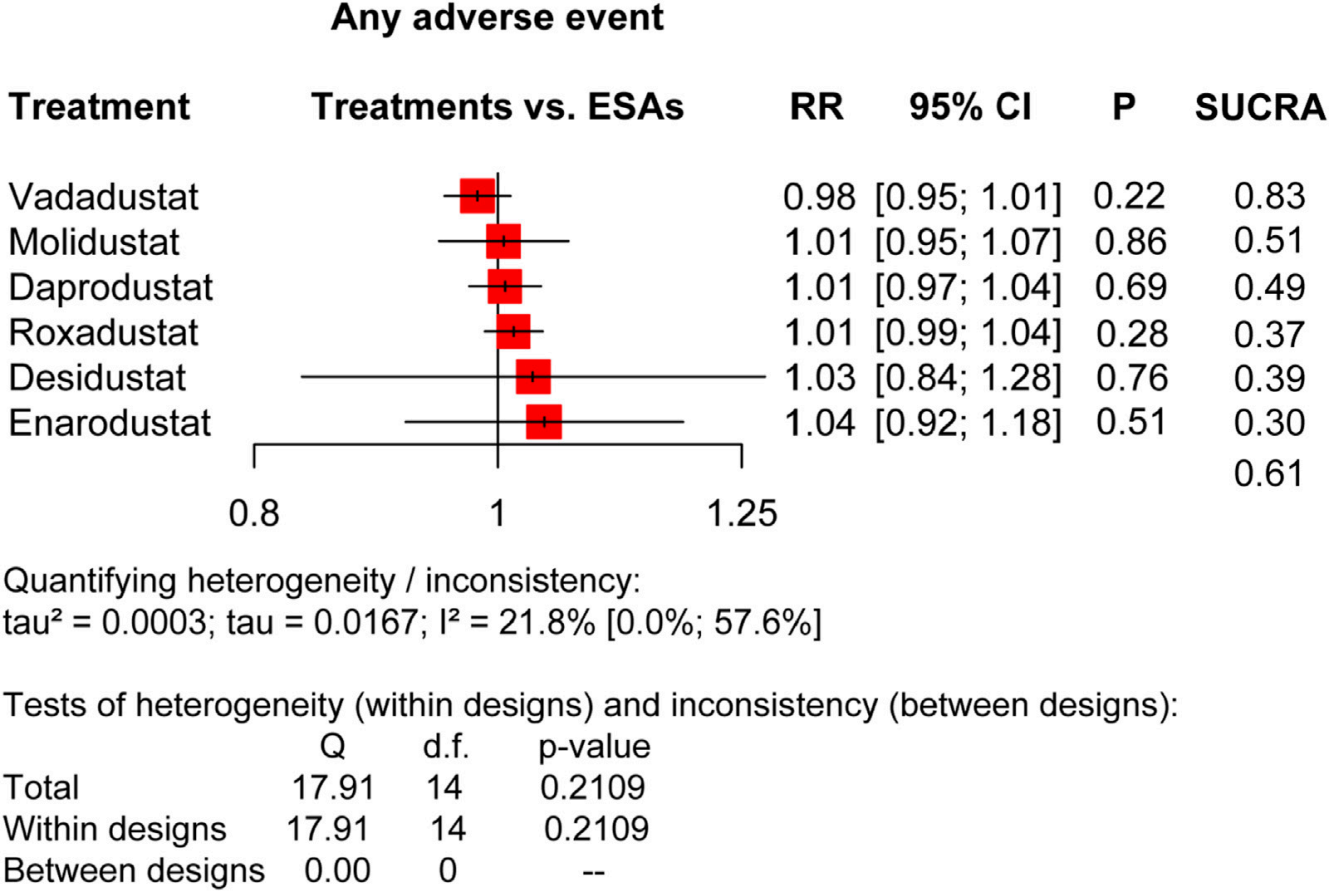
Forest plots for the safety of any adverse event. AEs, adverse events; ESAs, erythropoiesis-stimulating agents; RR, risk ratio; 95% CI, 95% confidence interval; and SUCRA, surface under the cumulative ranking.

**FIGURE 3:**
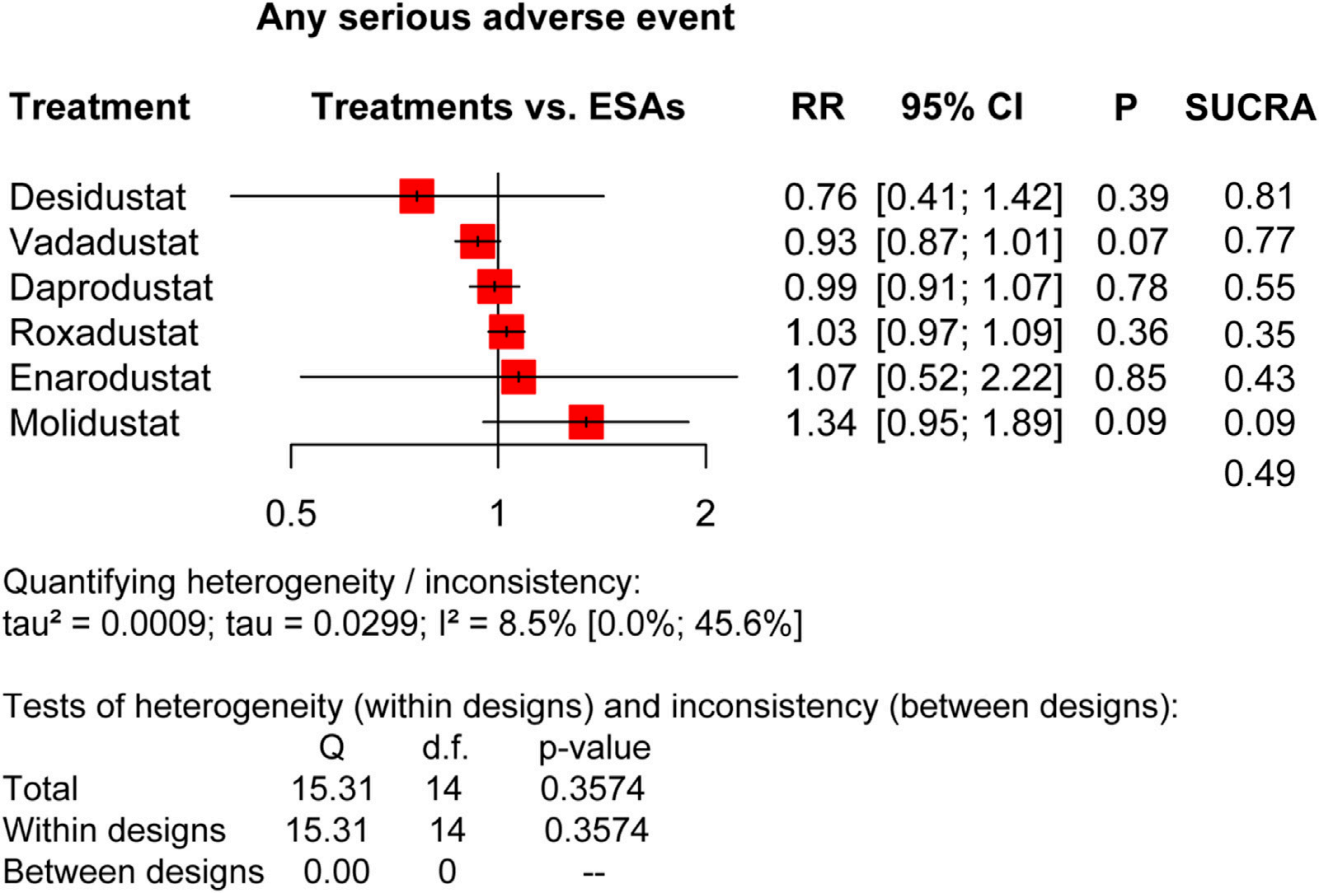
Forest plots for the safety of any serious adverse event. SAEs, serious adverse events; ESAs, erythropoiesis-stimulating agents; RR, risk ratio; 95% CI, 95% confidence interval; and SUCRA, surface under the cumulative ranking.

**FIGURE 4:**
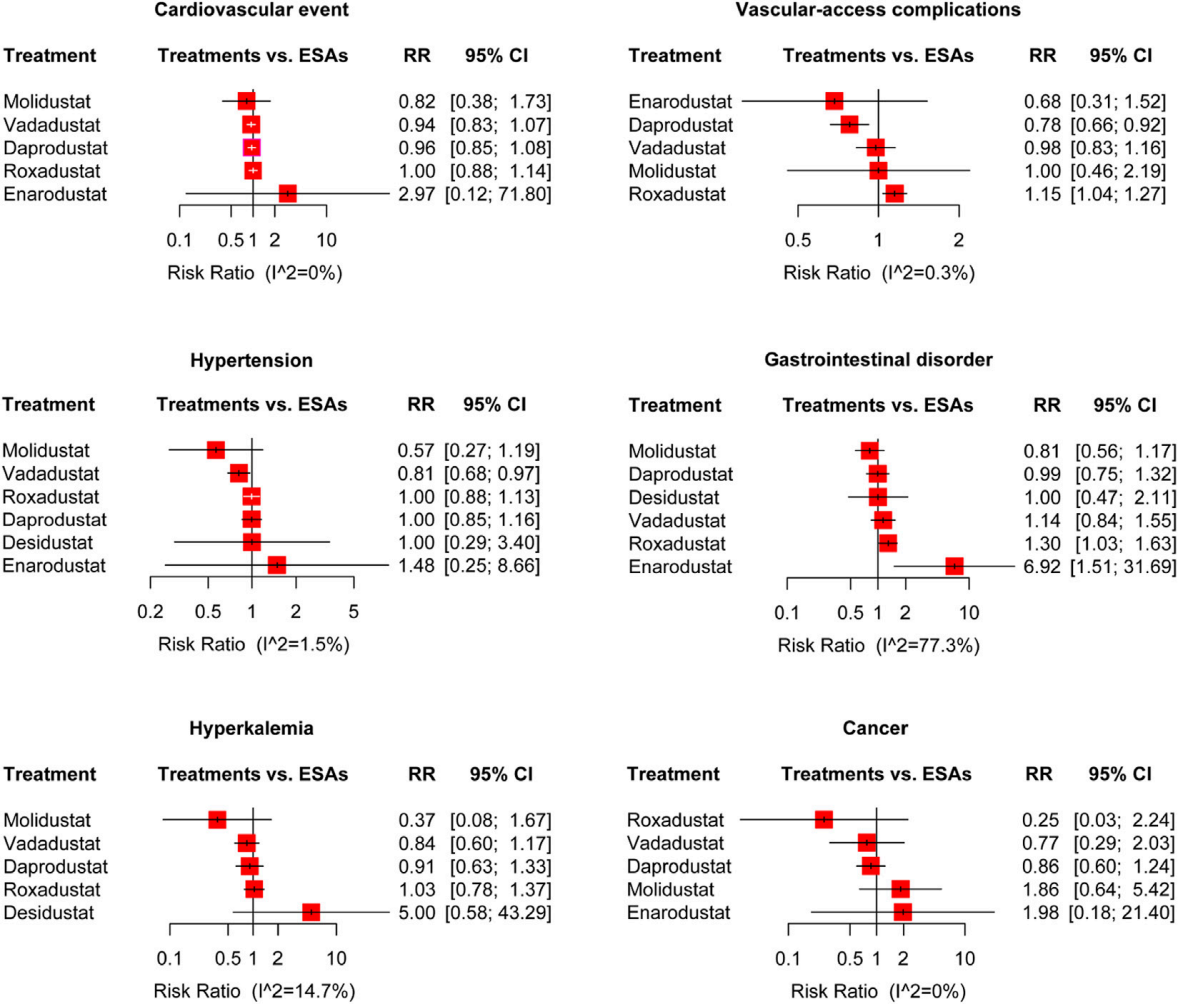
Forest plots for the safety of the treatment-emergent adverse events and the risk ratio of cardiovascular events, vascular-access complications, hypertension, gastrointestinal disorder, hyperkalemia, and cancer. ESAs, erythropoiesis-stimulating agents; RR, risk ratio; and 95% CI, 95% confidence interval.

**FIGURE 5:**
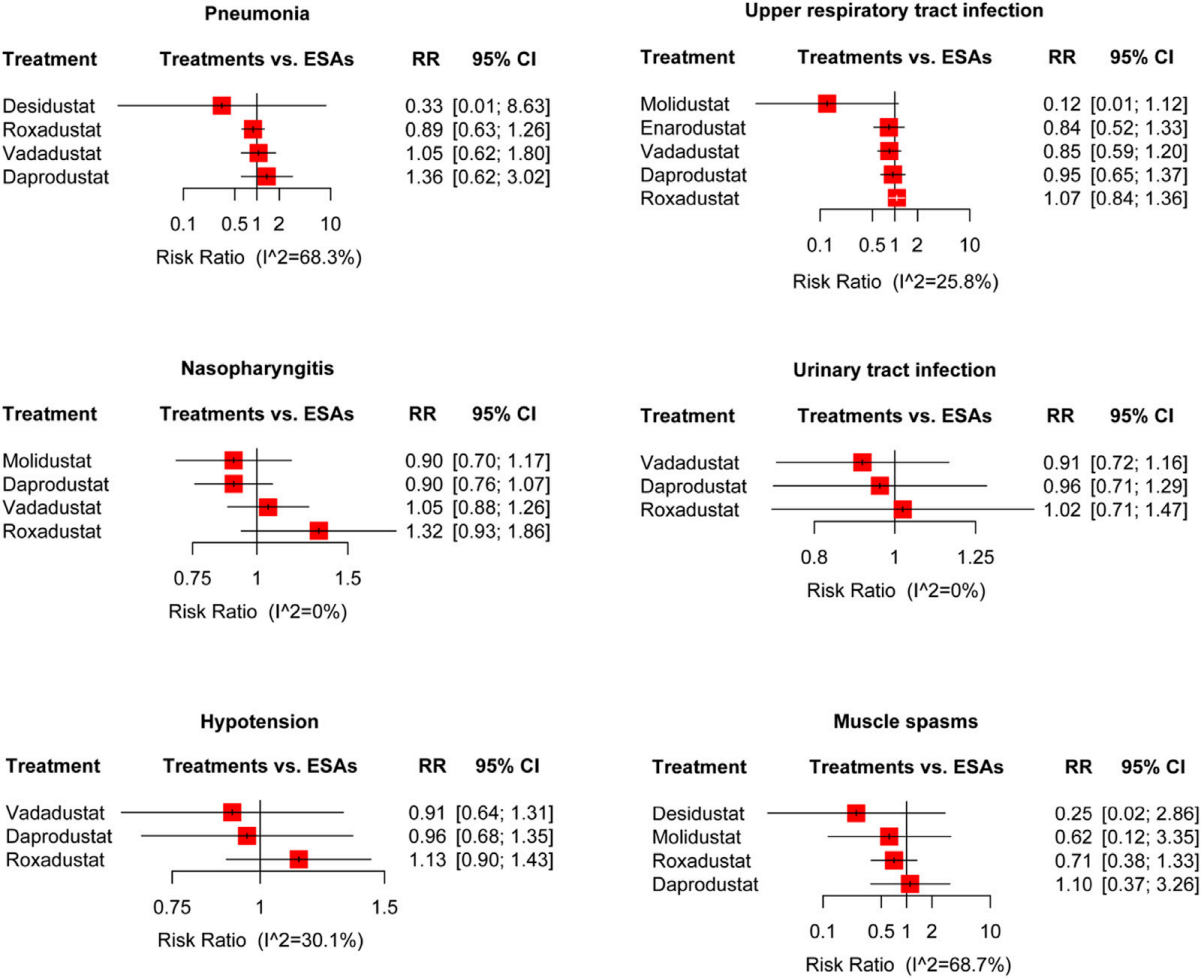
Forest plots for the safety of the treatment-emergent adverse events. The risk ratio of pneumonia, upper respiratory tract infection, nasopharyngitis, urinary tract infection, hypotension, and muscle spasms are displayed separately. ESAs, erythropoiesis-stimulating agents; RR, risk ratio; and 95% CI, 95% confidence interval.

We compared HIF-PHIs with ESAs based on major treatment-emergent adverse event (TEAE) risks, including AEs and SAEs, as the pooled results presented in Figures 2, 3, and relative risks for specific adverse events in DD CKD patients are shown in Figures 4, 5.

#### 3.4.1 AEs and SAEs

In terms of total AEs (20 trials) (Figure 2), it was indicated that the overall performance of each agent was not different, as roxadustat (RR: 1.01, 95% CI: 0.99–1.04, *p* = 0.28, I^2^ = 21.8%), enarodustat (RR: 1.04, 95% CI: 0.92–1.18, *p* = 0.51, I^2^ = 21.8%), daprodustat (RR: 1.01, 95% CI: 0.97–1.04, *p* = 0.69, I^2^ = 21.8%), desidustat (RR: 1.03, 95% CI:0.84–1.28, *p* = 0.76, I^2^ = 21.8%), vadadustat (RR: 0.98, 95% CI: 0.95–1.01, *p* = 0.22, I^2^ = 21.8%), and molidustat (RR: 1.01, 95% CI: 0.95–1.07, *p* = 0.86, I^2^ = 21.8%), and the SUCRA scores were vadadustat (0.83), ESAs (0.61), molidustat (0.51), daprodustat (0.49), desidustat (0.39), roxadustat (0.37), and enarodustat (0.30). In terms of total SAEs (20 trials) (Figure 3), there was no apparent difference between the agents, but the SUCRA rankings were not entirely consistent with that of AEs, which were desidustat (0.81), vadadustat (0.77), daprodustat (0.55), ESAs (0.49), enarodustat (0.43), roxadustat (0.35), and molidustat (0.09).

#### 3.4.2 Pairwise comparisons and net ranking of safety

As shown in Table 2, there were no significant differences in AEs and SAEs between each agent of HIF-PHIs, according to the pairwise comparisons. The intuitive displays of net ranking are shown in Supplementary Figures S10–S13.

**TABLE 2:** Pairwise comparisons of the safety of AEs (green) and SAEs (orange).

Roxadustat	0.96 (0.46, 1.99) *p* = 0.91	1.04 (0.94, 1.15) *p* = 0.44	1.35 (0.72, 2.52) *p* = 0.35	1.03 (0.97, 1.09) *p* = 0.36	1.10 (1.00, 1.21) *p* = 0.05	0.77 (0.54, 1.09) *p* = 0.14
0.97 (0.85, 1.11) *p* = 0.67	Enarodustat	1.08 (0.52, 2.25) *p* = 0.83	1.41 (0.54, 3.66) *p* = 0.49	1.07 (0.52, 2.22) *p* = 0.85	1.15 (0.55, 2.38) *p* = 0.71	0.80 (0.36, 1.79) *p* = 0.59
1.01 (0.97, 1.05) *p* = 0.72	1.04 (0.91, 1.18) *p* = 0.59	Daprodustat	1.30 (0.69, 2.43) *p* = 0.42	0.99 (0.91, 1.07) *p* = 0.78	1.06 (0.95, 1.18) *p* = 0.32	0.74 (0.52, 1.05) *p* = 0.09
0.98 (0.79, 1.22) *p* = 0.87	1.01 (0.79, 1.29) *p* = 0.94	0.97 (0.79, 1.21) *p* = 0.81	Desidustat	0.76 (0.41, 1.42) *p* = 0.39	0.82 (0.44, 1.53) *p* = 0.52	0.57 (0.28, 1.16) *p* = 0.12
1.01 (0.09, 1.04) *p* = 0.006	1.04 (0.92, 1.18) *p* = 0.379	1.01 (0.97, 1.04) *p* = 0.69	1.03 (0.84, 1.28) *p* = 0.76	ESAs	1.07 (0.99, 1.15) *p* = 0.07	0.75 (0.53, 1.05) *p* = 0.09
1.03 (0.99, 1.08) *p* = 0.11	1.06 (0.93, 1.21) *p* = 0.058	1.03 (0.98, 1.07) *p* = 0.26	1.05 (0.85, 1.30) *p* = 0.64	1.02 (0.99, 1.05) *p* = 0.22	Vadadustat	0.70 (0.49, 0.99) *p* = 0.04
1.01 (0.95, 1.08) *p* = 0.78	1.04 (0.90, 1.19) *p* = 0.60	1.00 (0.94, 1.07) *p* = 0.97	1.03 (0.82, 1.28) *p* = 0.81	0.99 (0.94, 1.06) *p* = 0.86	0.98 (0.91, 1.04) *p* = 0.48	Molidustat

The RRs and 95% CI are represented by the data in each grid, evaluated between agents from top-left to bottom-right. The blue areas represent the agents, the green areas represent the safety of AEs (RR and 95% CI) between different agents, and the orange areas represent the safety of SAEs (RR and 95% CI). AEs, adverse events; SAEs, serious adverse events; ESAs, erythropoiesis-stimulating agents; RR, risk ratio; 95% CI, 95% confidence interval.

#### 3.4.3 Subgroup analysis of safety

The influence of mean age, sex ratio, and duration of treatment was investigated (Supplementary Figures S14, S15). As shown in Supplementary Tables S1, S2, the SUCRA score rankings were directly consistent across the subgroups.

#### 3.4.4 Cardiovascular events

We analyzed the risk of cardiovascular events of HIF-PHIs in 17 trials, including 13,492 participants (Akizawa et al., 2019; Chen et al., 2019; Macdougall et al., 2019; Meadowcroft et al., 2019; Akizawa et al., 2020a; Akizawa et al., 2020b; Akizawa et al., 2021a; Charytan et al., 2021; Eckardt et al., 2021; Nangaku et al., 2021; Provenzano et al., 2021; Singh et al., 2021; Coyne et al., 2022; Fishbane et al., 2022; Hou et al., 2022; Singh et al., 2022). No obviously increased RR was indicated in DD patients with roxadustat (RR: 1.00, 95% CI: 0.88–1.14, *p* = 0.97, I^2^ = 0%), enarodustat (RR: 2.97, 95% CI: 0.12–71.80, *p* = 0.50, I^2^ = 0%), daprodustat (RR: 0.96, 95% CI: 0.85–1.08, *p* = 0.48, I^2^ = 0%), vadadustat (RR: 0.94, 95% CI: 0.83–1.07, *p* = 0.35, I^2^ = 0%), and molidustat (RR: 0.82, 95% CI: 0.38–1.73, *p* = 0.60, I^2^ = 0%) compared with the ESAs. The SUCRA rankings were molidustat (0.70), vadadustat (0.66), daprodustat (0.59), roxadustat (0.42), ESAs (0.40), and enarodustat (0.24) (Figure 4). The trials of molidustat and desidustat were excluded because neither total nor scattered cardiovascular events were reported (Akizawa et al., 2021b; Gang et al., 2022).

#### 3.4.5 Vascular-access complication

Sixteen trials of five agents of HIF-PHIs reported vascular-access complications of HIF-PHIs for DD CKD anemia, containing 13,915 participants (Akizawa et al., 2019; Chen et al., 2019; Meadowcroft et al., 2019; Akizawa et al., 2020a; Akizawa et al., 2021a; Akizawa et al., 2021b; Charytan et al., 2021; Csiky et al., 2021; Eckardt et al., 2021; Nangaku et al., 2021; Provenzano et al., 2021; Singh et al., 2021; Coyne et al., 2022; Fishbane et al., 2022). Compared with ESAs, daprodustat performed much better in the risk of vascular-access complication (RR: 0.78, 95% CI: 0.66–0.92, *p* <0.01, I^2^ = 0.3%), while roxadustat performed a little worse (RR: 1.15, 95% CI: 1.04–1.27, *p* <0.01, I^2^ = 0.3%). The pooled results showed no significant difference in enarodustat, vadadustat, and molidustat (Figure 4).

#### 3.4.6 Hypertension

The risk ratios of hypertension (17 trials of six HIF-PHIs, containing 14,094 participants (Akizawa et al., 2019; Chen et al., 2019; Macdougall et al., 2019; Meadowcroft et al., 2019; Akizawa et al., 2020b; Akizawa et al., 2021a; Charytan et al., 2021; Csiky et al., 2021; Eckardt et al., 2021; Provenzano et al., 2021; Singh et al., 2021; Coyne et al., 2022; Fishbane et al., 2022; Gang et al., 2022; Hou et al., 2022; Singh et al., 2022)) were statistically lower in the vadadustat (RR: 0.81, 95% CI: 0.68–0.97, *p* = 0.02, I^2^ = 1.5%), and no remarkably increase risk in roxadustat, enarodustat, daprodustat, desidustat and Molidustat, compared with ESAs (Figure 4).

#### 3.4.7 Gastrointestinal disorder

With DD patients for gastrointestinal disorder (20 trials), the pooled results showed a statistical increase in roxadustat (RR: 1.30, 95% CI: 1.03–1.63, *p* = 0.03, I^2^ = 77.3%) and enarodustat (RR: 6.92, 95% CI: 1.51–31.69, *p* = 0.01, I^2^ = 77.3%), but no significant differences in daprodustat, desidustat, vadadustat, and molidustat (Figure 4).

#### 3.4.8 Hyperkalemia

In hyperkalemia, 13 trials of five agents of HIF-PHIs were reported, containing 12,771 participants (Chen et al., 2019; Meadowcroft et al., 2019; Akizawa et al., 2021b; Charytan et al., 2021; Eckardt et al., 2021; Nangaku et al., 2021; Provenzano et al., 2021; Singh et al., 2021; Coyne et al., 2022; Fishbane et al., 2022; Gang et al., 2022; Hou et al., 2022). There was no significant increase in risks with roxadustat (RR: 1.03, 95% CI: 0.78–1.37, *p* = 0.81, I^2^ = 14.7%), daprodustat (RR: 0.91, 95% CI: 0.63–1.33, *p* = 0.63, I^2^ = 14.7%), desidustat (RR: 5.00, 95% CI: 0.58–43.29, *p* = 0.14, I^2^ = 14.7%), vadadustat (RR: 0.84, 95% CI: 0.60–1.17, *p* = 0.30, I^2^ = 14.7%), and molidustat (RR: 0.37, 95% CI: 0.08–1.67, *p* = 0.20, I^2^ = 14.7%) compared with ESAs (Figure 4).

#### 3.4.9 Cancer

The RRs of cancer (nine trials of five HIF-PHIs, containing 5,189 participants (Meadowcroft et al., 2019; Akizawa et al., 2020a; Akizawa et al., 2020b; Akizawa et al., 2021a; Akizawa et al., 2021b; Nangaku et al., 2021; Singh et al., 2021; Coyne et al., 2022; Singh et al., 2022)) with HIF-PHIs in DD patients showed no statistical increases compared with ESAs: roxadustat (RR: 0.25, 95% CI: 0.03–2.24, *p* = 0.22, I^2^ = 0%), enarodustat (RR: 1.98, 95% CI: 0.18–21.40, *p* = 0.57, I^2^ = 0%), daprodustat (RR: 0.86, 95% CI: 0.60–1.24, *p* = 0.42, I^2^ = 0%), vadadustat (RR: 0.77, 95% CI: 0.30–2.03, *p* = 0.60, I^2^ = 0%), and molidustat (RR: 1.86, 95% CI: 0.64–5.42, *p* = 0.25, I^2^ = 0%) (Figure 4).

#### 3.4.10 Pneumonia and upper respiratory tract infection

The results indicated no increased risk of pneumonia (11 trials of four HIF-PHIs, containing 12,985 participants (Chen et al., 2019; Akizawa et al., 2020b; Charytan et al., 2021; Csiky et al., 2021; Eckardt et al., 2021; Provenzano et al., 2021; Singh et al., 2021; Coyne et al., 2022; Fishbane et al., 2022; Gang et al., 2022)) with roxadustat, daprodustat, desidustat, and vadadustat, compared with ESAs. In terms of upper respiratory tract infection among DD patients (12 trials of five HIF-PHIs, containing 12,037 patients (Chen et al., 2019; Akizawa et al., 2021a; Akizawa et al., 2021b; Charytan et al., 2021; Csiky et al., 2021; Eckardt et al., 2021; Nangaku et al., 2021; Singh et al., 2021; Fishbane et al., 2022; Hou et al., 2022; Singh et al., 2022)), there was no significant difference between HIF-PHIs and ESAs, consisting of roxadustat, enarodustat, daprodustat, vadadustat, and molidustat (Figure 5).

#### 3.4.11 Nasopharyngitis and urinary tract infection

Ten trials reported the nasopharyngitis AEs of four HIF-PHIs for anemia, containing 8,732 participants (Macdougall et al., 2019; Meadowcroft et al., 2019; Akizawa et al., 2020a; Akizawa et al., 2021b; Eckardt et al., 2021; Nangaku et al., 2021; Singh et al., 2021; Singh et al., 2022). The results indicated no statistical difference in the risk of roxadustat, daprodustat, vadadustat, and molidustat compared to ESAs. Seven trials of three HIF-PHIs reported urinary tract infection AEs, containing 11,078 participants (Flamme et al., 2014; Charytan et al., 2021; Eckardt et al., 2021; Provenzano et al., 2021; Singh et al., 2021; Fishbane et al., 2022), and no increased risks were showed between HIF-PHIs (roxadustat, daprodustat, and vadadustat) and ESAs (Figure 5).

#### 3.4.12 Hypotension and muscle spasms

The pooled results indicated no significant difference in hypotension (11 trials of three HIF-PHIs, containing 12,763 participants (Chen et al., 2019; Charytan et al., 2021; Csiky et al., 2021; Eckardt et al., 2021; Provenzano et al., 2021; Singh et al., 2021; Coyne et al., 2022; Fishbane et al., 2022; Hou et al., 2022; Singh et al., 2022)) with roxadustat, daprodustat, and vadadustat, compared to ESAs. Focusing on muscle spasms, no increased risk was reported (nine trials of four HIF-PHIs, containing 4,255 participants (Chen et al., 2019; Akizawa et al., 2020b; Akizawa et al., 2021b; Charytan et al., 2021; Csiky et al., 2021; Provenzano et al., 2021; Gang et al., 2022; Hou et al., 2022; Singh et al., 2022)) between HIF-PHIs (roxadustat, daprodustat, desidustat, and molidustat) and ESAs (Figure 5).

### 3.5 Efficacy endpoint assessment

#### 3.5.1 Response rate of Hb

**FIGURE 6:**
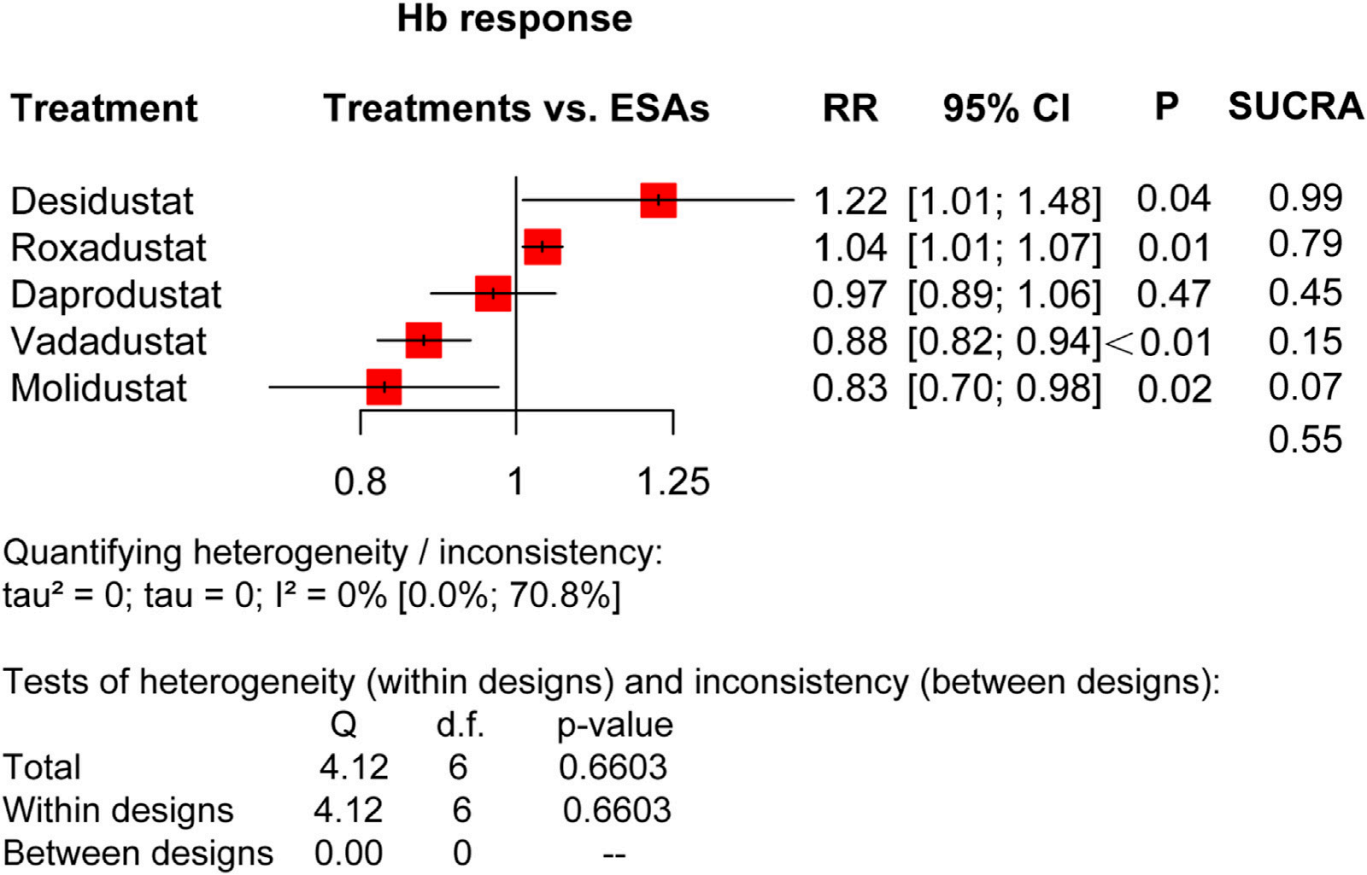
Forest plots for the efficacy of Hb response. ESAs, erythropoiesis-stimulating agents; RR, risk ratio; 95% CI, 95% confidence interval; and SUCRA, surface under the cumulative ranking.

Reporting 11 trials of five types of HIF-PHIs in 8,167 DD CKD patients (Chen et al., 2019; Akizawa et al., 2020a; Akizawa et al., 2020b; Akizawa et al., 2021b; Charytan et al., 2021; Csiky et al., 2021; Eckardt et al., 2021; Provenzano et al., 2021; Gang et al., 2022; Hou et al., 2022), our network meta-analysis showed significant increases in Hb response (defined as ΔHb≥1.0 g/dL and Hb level≥11.0 g/dL) in roxadustat (RR: 1.04, 95% CI: 1.01–1.07, *p* <0.01, I^2^ = 0%) and desidustat (RR: 1.22, 95% CI: 1.01–1.48, *p* = 0.04, I2 = 0%), compared with ESAs, whereas noticeable reductions were indicated in vadadustat (RR: 0.88, 95% CI: 0.82–0.94, *p* <0.01) and molidustat (RR: 0.83, 95% CI: 0.70–0.98, *p* = 0.02, I^2^ = 0%). There was no significant difference between daprodustat and ESAs (RR: 0.97, 95% CI: 0.89–1.06, *p* = 0.47, I^2^ = 0%). The SUCRA net rankings were desidustat (0.99), roxadustat (0.79), ESAs (0.55), daprodustat (0.45), vadadustat (0.15), and molidustat (0.07) (Figure 6).

#### 3.5.2 Mean change in the hemoglobin level from baseline

Regarding ΔHb, this network meta-analysis included 20 trials involving six HIF-PHIs (Supplementary Figure S16). Substantial heterogeneity (I2 = 74.6%) led to choosing random-effects models in the following analyses. A significant increase was found in efficacy between roxadustat and ESAs (MD:0.19 g/dL, 95% CI:0.07–0.30, *p* <0.01), and no statistic differences were found with enarodustat, daprodustat, desidustat, vadadustat, and molidustat. Since the network structure for DD CKD patients contains no loop, the full design-by-treatment interaction random-effects model is not applied. Moreover, the net-rankings were roxadustat (0.88), desidustat (0.72), enarodustat (0.60), daprodustat (0.56), ESAs (0.46), vadadustat (0.23), and molidustat (0.05).

#### 3.5.3 Pairwise comparisons of efficacy

As shown in Supplementary Table S3, for Hb response, roxadustat and desidustat performed better than vadadustat (RR: 1.18, 95% CI: 1.10–1.27, *p* <0.01; RR: 1.40, 95% CI: 1.14–1.71, *p* = 0.001) and molidustat (RR: 1.25, 95% CI: 1.06–1.48, *p* = 0.01; RR: 1.48, 95% CI: 1.15–1.90, *p* = 0.002). Daprodustat performed as well as desidustat (RR: 0.79, 95% CI: 0.64–0.98, *p* = 0.03). There was no significant difference between the other agents.

#### 3.5.4 Subgroup analysis of efficacy

The influence of mean age, sex ratio, and duration of treatment was investigated (Supplementary Figure S17). As shown in Supplementary Table S4, the SUCRA score rankings were directly consistent across the subgroups.

#### 3.5.5 Network meta-analysis of iron metabolism indicators

Eight trials in DD CKD patients with a total of 16 arms reported the influence of these agents on hepcidin. Results revealed a significant reduction in Δhepcidin with roxadustat (MD: 13.11 μg/L, 95% CI: 24.65–1.57, *p* = 0.03, I^2^ = 83%) and no statistical effects with vadadustat and molidustat compared with ESAs. The efficacy of HIF-PHIs on ΔTSAT, Δserum iron, ΔFerritin, and ΔTIBC is shown in Supplementary Figure S18.

### 3.6 Small-study safety and effect analysis

The funnel plots comparing the safety of AEs and SAEs, the efficacy of Hb response, and ΔHb showed a symmetrical pattern in DD CKD patient groups (Supplementary Figures S19–S22), which indicated that there might not be a statistical small-study effect (Egger’s test; *p* >0.05).

### 3.7 Sensitivity analysis

Although the primary analysis was based on a frequentist model, it was repeated with a Bayesian model to test the robustness. The method showed similar SUCRA scores.

## 4 Discussion

This network meta-analysis aims to assess the safety and efficacy of different agents containing HIF-PHIs for treating anemia in DD CKD patients. Safety and efficacy data for HIF-PHIs are drawn from 20 trials comparing the HIF-PHI agent with ESA controls in anemic DD patients. By summarizing their pairwise comparison and overall safety and efficacy, results showed that all six HIF-PHIs did not increase the risk of any adverse events and serious adverse events compared to ESAs. No notable differences were found in this network meta-analysis in the risk of cardiovascular events, hyperkalemia, cancer, pneumonia, upper respiratory tract infection, nasopharyngitis, urinary tract infection, hypotension, and muscle spasms between HIF-PHIs and ESAs. It is worth mentioning that roxadustat and enarodustat were associated with a statistical increase in the risk of gastrointestinal disorder. In the risk of vascular-access complication, roxadustat performed worse with more risks than ESAs. However, compared to ESAs, there was a lower risk of vascular-access complications in daprodustat and hypertension in vadadustat. In terms of efficacy, compared with ESAs in Hb response, our meta-analysis showed significant increases in roxadustat and desidustat, whereas noticeable reductions in vadadustat and molidustat. There were no significant differences indicated between daprodustat ESAs.

ESAs are widely taken in the remedy of anemic patients. Studies have shown that they can promote the proliferation of erythroid progenitors after erythropoietin receptor (EpoRs) binding (Thavarajah and Choi, 2019), imitating the action of endogenous erythropoietin to promote Hb synthesis effectively (Koury and Haase, 2015; Zheng et al., 2020). Based on the results presented, recent studies found that HIF-PHIs had therapeutic effects similar to ESAs without increasing significant adverse effects (Wang et al., 2020; Wen et al., 2020; Nangaku et al., 2021; Singh et al., 2022). HIF, an iron sensor and regulator, is a pharmacological approach that can enhance intestinal iron uptake and transport by imitating coordination of erythropoiesis and iron metabolism in response to hypoxia, providing a balanced physiological method of the treatment of renal anemia (Peyssonnaux et al., 2008; Koury and Haase, 2015; Haase, 2021). PHD enzymes, as dioxygenases, can prevent the formation of HIF transcription factors (Huang et al., 2002). HIF-PHIs, potent reversible inhibitors of all PHD isoforms (Arezes et al., 2018), correct and maintain hemoglobin levels in CKD patients by activating the HIF oxygen-sensing pathway. HIF-PHIs promote erythropoiesis by increasing the production of endogenous erythropoietin, reducing hepcidin levels, and regulating iron metabolism (Haase, 2021). Therefore, HIF-PHIs have broad therapeutic potential for the remedy for renal anemia, and a reduction in intravenous iron supplementation replacement may result from this.

Our study compared the efficacy of six types of HIF-PHIs with ESAs in treating DD CKD patients with anemia, focusing on their safety on AEs, SAEs, and 12 common adverse events. Our results show that the overall risk of HIF-PHIs is not higher than ESAs in patients on dialysis, and the safety ranking of each agent in AEs is inconsistent with that in SAEs. When specific to a particular adverse reaction like cardiovascular events and gastrointestinal disorder, HIF-PHIs performed varied but generally as well as ESAs. For efficacy, we focused on Hb response, ΔHb, and iron metabolism. About increasing the Hb response and ΔHb, each agent of HIF-PHIs showed different performance and no significant difference compared to ESAs. Moreover, the influence of mean age, sex ratio, and duration of treatment was investigated, and the SUCRA score rankings were directly consistent across the subgroups. These pieces of evidence support that HIF-PHIs have promising therapeutic effects and can be extended to clinical application. And to increase the strength of the relevant study, more extensive, high-quality research, including, but not limited to, Enarodustat and Desidustat, is demanded further to confirm the efficacy and safety of these medicines.

HIF-PHIs have been compared and analyzed in the published literature with ESAs, including Roxadustat, Daprodusta, Molidustat, Vadadustat, Enarodustat, and Desidustat. Some meta-analyses concluded that Roxadustast increases the ΔHb and reduces hepcidin in either DD or NDD patients (Liu et al., 2020; Liu et al., 2021). Daprodustat may better influence DD CKD patients’ optimizing iron metabolism (Fu et al., 2022) and is not inferior to ESAs regarding ΔHb and cardiovascular diseases (Singh et al., 2021). Nevertheless, some literature proposed that safety data of HIF-PHIs like Roxadustat is still emerging, and attention must be poured into the risk of TEAE, especially SAEs during therapy (Liu et al., 2020; Zheng et al., 2022). The abovementioned research is basically limited to studying a specific drug in HIF-PHI, and the safety is still controversial. In addition, some overall analyses of HIF-PHIs show that HIF-PHIs improve renal anemia and correct iron metabolism in a short time without increasing the occurrence rate of AEs and SAEs (Wen et al., 2020), or HIF-PHIs are effective and relatively well tolerated in renal anemia patients without dialysis (Zheng et al., 2020). However, these studies mainly focus on the overall analysis of the HIF-PHIs of AEs and SAEs instead of the risk comparison of specific adverse events, not to mention the direct comparison of different agents of HIF-PHIs.

HIF-PHIs hold great promise for the treatment and management of renal anemia patients. Although their safety is not inferior to current clinical agents like ESAs, attention should still be paid to possible problems surrounding their use. On the one hand, it is well known that many unrelated genes are regulated by HIF during erythropoiesis and regulation, leading to some potential adverse effects, which are currently unknown. For instance, HIF-PHIs may increase the risk of gastrointestinal bleeding because HIF can regulate abnormal angiogenesis in the gastrointestinal tract by directly targeting the VEGF pathway (Feng et al., 2016). Also, via the treatment of HIF-PHIs, the consequences of maintaining physiologic levels of endogenous EPO and the cardiovascular effects of normalizing Hb levels have not been established in the clinical trials of HIF-PHI that we have learned to date. These require further research to elucidate.

This study has some advantages over previous related studies but also has its limitations. First, it is a network meta-analysis comparing different agents of HIF-PHIs treating DD anemic CKD patients, which mainly focuses on and analyzes the safety, providing evidence for the clinical use of HIF-PHIs. Based on direct and circumstantial evidence, we offered a comprehensive net-ranking and pairwise comparison of the safety of these agents of AEs and SAEs in two groups and conducted a comparative study of each agent for specific TEAEs, including cardiovascular diseases. At the same time, we made an overall ranking analysis and pairwise comparison of Hb response and ΔHb, and conducted subgroup analyses of the influence of mean age, sex, and duration of treatment. By sensitivity analysis, the similar SUCRA scores of frequentist and Bayesian models increased our findings’ confidence. All these provide a research basis and reference value for guiding the use of clinical drugs and promoting the clinical application of new medicines. Also, this study had some limitations. First, the results were expected to be clarified by further research because of the small sample size of included studies and the large 95% CI width, yet a wide geographical and demography spread of study settings increases the generalizability of the study findings. Second, the doses of HIF-PHI given in the treatment groups varied across studies, as did the duration of treatment. Because there was no head-to-head comparison, the results compared in our meta-analysis need to be further confirmed by future data.

## 5 Conclusion

Although the overall AEs and SAEs of HIF-PHIs were not significantly different from ESAs, observed statistical differences in individual HIF-PHIs with higher risks of gastrointestinal disturbances and vascular-access complications and lower risk of hypertension from ESAs warranted attention in clinical application. Moreover, as HIF-PHI is a kind of emerging drug for the treatment of renal anemia, there is a lack of studies on its long-term efficacy and safety; for example, its effect on kidney function and the progression of kidney disease in patients remains unknown. Also, some studies have indicated that HIF-PHIs can show antihypertensive effects in a model of CKD and protect endothelial cells (Flamme et al., 2014). All these need to be further verified in more clinical human studies.

## Data Availability

The original contributions presented in the study are included in the article/Supplementary Material; further inquiries can be directed to the corresponding authors.
