# Supplementary material for: Safety of HIF prolyl hydroxylase inhibitors for anemia in dialysis patients: a systematic review and network meta-analysis
Source: Front Pharmacol. 2023 May 24;14:1163908. doi: 10.3389/fphar.2023.1163908 (PMC10244523; doi:10.3389/fphar.2023.1163908)
Supplement: Supplementary file 2 [file DataSheet1.docx]

**Safety of HIF Prolyl-Hydroxylase Inhibitors for Anemia in Dialysis Patients: A Systematic Review and Network Meta-Analysis**

**Network Meta-Analysis of HIF-PHIs Safety**

**Dinghua Chen^1^, Yue Niu^1^, Fei Liu^2^, Yue Yang^1^, Xue Wang^1^, Ping Li^1*^, Xiangmei Chen^1*^**

^1^Department of Nephrology, First Medical Center of Chinese PLA General Hospital, Beijing, China

^2^Department of Urology, Chinese Academy of Medical Sciences and Peking Union Medical College, Beijing, China

***Correspondence:**

Ping Li, E-mail: liping@301hospital.com.cn

Xiangmei Chen, E-mail: xmchen301@126.com

Department of Nephrology,

Chinese PLA Institute of Nephrology,

State Key Laboratory of Kidney Disease,

National Clinical Research Center for Kidney Disease,

Chinese PLA General Hospital, Chinese PLA Medical School,

Fuxing Road 28, Beijing 100853, P.R. China

Phone: 86-10-66935462

Fax: 86-10-68130297

**SUPPLEMENTARY MATERIAL**

**Supplementary Material S1.** PubMed search strategy.

**Supplementary Table S1.** Subgroup SUCRAs of any adverse event.

**Supplementary Table S2.** Subgroup SUCRAs of any serious adverse event.

**Supplementary Table S3.** Pairwise comparisons of Hb response (green) and ΔHb (orange).

**Supplementary Table S4.** Subgroup SUCRAs of Hb response.

**Supplementary Figure S1.** Structure of network formed by treatments.

**Supplementary Figure S2.** Risk of bias domains of any adverse event summary of included trials.

**Supplementary Figure S3.** Risk of bias domains of any adverse event summary of included trials.

**Supplementary Figure S4.** Risk of bias domains of any serious adverse event summary of included trials.

**Supplementary Figure S5.** Risk of bias domains of any serious adverse event summary of included trials.

**Supplementary Figure S6.** Risk of bias domains of Hb response summary of included trials for DD CKD patients.

**Supplementary Figure S7.** Risk of bias domains of Hb response summary of included trials for DD CKD patients.

**Supplementary Figure S8.** Risk of bias domains of ΔHb summary of included trials.

**Supplementary Figure S9.** Risk of bias domains of ΔHb summary of included trials.

**Supplementary Figure S10.** Net-ranking for the safety of any adverse event.

**Supplementary Figure S11.** Net-ranking for the safety of any serious adverse event.

**Supplementary Figure S12.** Net-ranking for the Hb response.

**Supplementary Figure S13.** Net-ranking for the ΔHb.

**Supplementary Figure S14.** Forest plots for the influence on any adverse event of mean age, male ratio, and duration of treatment.

**Supplementary Figure S15.** Forest plots for the influence on any serious adverse event of mean age, male ratio, and duration of treatment.

**Supplementary Figure S16.** Forest plots for the ΔHb.

**Supplementary Figure S17.** Forest plots for the influence on Hb response of mean age, male ratio, and duration of treatment.

**Supplementary Figure S18.** Forest plots for iron metabolism indicators.

**Supplementary Figure S19.** Comparison-adjusted funnel plots for any adverse event.

**Supplementary Figure S20.** Comparison-adjusted funnel plots for any serious adverse event.

**Supplementary Figure S21.** Comparison-adjusted funnel plots for the Hb response.

**Supplementary Figure S22.** Comparison-adjusted funnel plots for the ΔHb.

**Supplementary Material S1**

PubMed search strategy

#1 ("Renal Insufficiency, Chronic"[Mesh]) OR ((((((((((((((((((((Chronic Renal Insufficiencies) ) OR (Renal Insufficiencies, Chronic)) OR (Chronic Renal Insufficiency)) OR (Kidney Insufficiency, Chronic)) OR (Chronic Kidney Insufficiency)) OR (Chronic Kidney Insufficiencies)) OR (Kidney Insufficiencies, Chronic)) OR (Chronic Kidney Diseases)) OR (Chronic Kidney Disease)) OR (Disease, Chronic Kidney)) OR (Diseases, Chronic Kidney)) OR (Kidney Disease, Chronic)) OR (Kidney Diseases, Chronic)) OR (Chronic Renal Diseases)) OR (Chronic Renal Disease)) OR (Disease, Chronic Renal)) OR (Diseases, Chronic Renal)) OR (Renal Disease, Chronic)) OR (Renal Diseases, Chronic))

#2 (((((hypoxia-inducible factor prolyl hydroxylase inhibitors [All Fields])) OR (HIF-PHIs)) OR (PHD inhibitor)) OR (hypoxia inducible factor stabilizer)) OR (HIF stabilizer) OR ((((roxadustat[All Fields])) OR (FG-4592)) OR (ASP1517)) OR (AZD9941) OR ((Vadadustat [All Fields])) OR (AKB-6548) OR ((Daprodustat [All Fields])) OR (GSK127883) OR ((Molidustat [All Fields])) OR (BAY 85-3934) OR ((Enarodustat [All Fields])) OR (JTZ-951) OR (DS-1093a [All Fields]) OR ((Desidustat [All Fields])) OR (ZYAN1) OR(JNJ-42905343 [All Fields]) OR (TP0463518 [All Fields]) OR (AKB-4924 [All Fields]) OR (DDO-3055 [All Fields]) OR (AKB-6899 [All Fields]) OR (HEC-53856 [All Fields]) OR (BGE-117 [All Fields]) OR (MK-8617 [All Fields]) OR (JNJ-42905343 [All Fields])

#3 #1 and #2

Filters: Randomized Controlled Trial, Humans, Adult: 18+ years, from 1000/1/1 - 2022/08/31

**Supplementary Table S1. Subgroup SUCRAs of any adverse event.**

| **Model** | **Subgroup** | **SUCRA** | | | | | | |
| --- | --- | --- | --- | --- | --- | --- | --- | --- |
|  |  | Daprodustat | Desidustat | Enarodustat | ESAs | Molidustat | Roxadustat | Vadadustat |
| Original model | - | 0.51 | 0.58 | 0.67 | 0.39 | 0.53 | 0.64 | 0.18 |
| Subgroup: Mean age | <60 | 0.50 | 0.58 | 0.67 | 0.39 | 0.53 | 0.64 | 0.20 |
|  | ≥60 | 0.49 | 0.58 | 0.67 | 0.40 | 0.54 | 0.63 | 0.20 |
| Subgroup: Male ratio | <0.6 | 0.41 | 0.54 | 0.63 | 0.50 | 0.55 | 0.69 | 0.17 |
|  | ≥0.6 | 0.47 | 0.56 | 0.65 | 0.36 | 0.58 | 0.70 | 0.18 |
| Subgroup: Duration of treatment | <52 weeks | 0.48 | 0.63 | 0.58 | 0.21 | 0.61 | 0.72 | 0.27 |
|  | 52 weeks | 0.38 | 0.61 | 0.55 | 0.52 | 0.56 | 0.69 | 0.18 |

ESAs: erythropoiesis-stimulating agents.

**Supplementary Table S2. Subgroup SUCRAs of any serious adverse event.**

| **Model** | **Subgroup** | **SUCRA** | | | | | | |
| --- | --- | --- | --- | --- | --- | --- | --- | --- |
|  |  | Daprodustat | Desidustat | Enarodustat | ESAs | Molidustat | Roxadustat | Vadadustat |
| Original model | - | 0.38 | 0.19 | 0.57 | 0.51 | 0.91 | 0.67 | 0.26 |
| Subgroup: Mean age | <60 | 0.44 | 0.16 | 0.59 | 0.56 | 0.89 | 0.64 | 0.22 |
|  | ≥60 | 0.50 | 0.17 | 0.60 | 0.40 | 0.90 | 0.68 | 0.24 |
| Subgroup: Male ratio | <0.6 | 0.34 | 0.18 | 0.51 | 0.58 | 0.91 | 0.71 | 0.27 |
|  | ≥0.6 | 0.37 | 0.19 | 0.53 | 0.48 | 0.92 | 0.73 | 0.28 |
| Subgroup: Duration of treatment | <52 weeks | 0.32 | 0.28 | 0.47 | 0.26 | 0.92 | 0.75 | 0.49 |
|  | 52 weeks | 0.22 | 0.25 | 0.43 | 0.61 | 0.91 | 0.72 | 0.36 |

ESAs: erythropoiesis-stimulating agents.

**Supplementary Table S3. Pairwise comparisons of the Hb response (green) and ΔHb (orange).**

| Roxadustat | 0.13 (-0.24, 0.49) p=0.49 | 0.15 (-0.04, 0.34) p=0.12 | 0.05 (-0.35, 0.44) p=0.82 | 0.19 (0.07, 0.30) p=0.002 | 0.30 (0.09, 0.52) p=0.01 | 0.48 (0.15, 0.82) p=0.01 |
| --- | --- | --- | --- | --- | --- | --- |
| — | Enarodustat | 0.02 (-0.35, 0.40) p=0.91 | -0.08 (-0.59, 0.43) p=0.76 | 0.06 (-0.28, 0.40) p=0.73 | 0.17 (-0.22, 0.56) p=0.38 | 0.36 (-0.11, 0.82) p=0.13 |
| 1.07 (0.98, 1.18) p=0.14 | — | Daprodustat | -0.10 (-0.51, 0.30) p=0.62 | 0.04 (-0.11, 0.19) p=0.63 | 0.15 (-0.08, 0.39) p=0.21 | 0.33 (-0.01, 0.68) p=0.06 |
| 0.85 (0.70, 1.03) p=0.10 | — | 0.79 (0.64, 0.98) p=0.03 | Desidustat | 0.14 (-0.24, 0.52) p=0.47 | 0.25 (-0.16, 0.67) p=0.23 | 0.44 (-0.05, 0.93) p=0.08 |
| 1.04 (1.01, 1.07) p=0.01 | — | 0.97 (0.89, 1.06) p=0.47 | 1.22 (1.01, 1.48) p=0.04 | ESAs | 0.11 (-0.07, 0.30) p=0.22 | 0.30 (-0.02, 0.61) p=0.06 |
| 1.18 (1.10, 1.27) p<0.001 | — | 1.10 (0.99, 1.23) p=0.08 | 1.40 (1.14, 1.71) p=0.001 | 1.14 (1.07, 1.22) p<0.001 | Vadadustat | 0.18 (-0.18, 0.54) p=0.33 |
| 1.25 (1.06, 1.48) p=0.01 | — | 1.17 (0.97, 1.41) p=0.10 | 1.48 (1.15, 1.90) p=0.002 | 1.21 (1.03, 1.42) p=0.02 | 1.06 (0.89, 1.26) p=0.53 | Molidustat |

The MDs, RRs and 95 % CI are represented by the data in each grid, evaluated between agents from top-left to bottom-right. The blue areas represent the agents, the green areas represent the effect on Hb response (RR and 95% CI) between different agents, and the orange areas represent the effect on ΔHb (MD and 95% CI). ΔHb: hemoglobin level changed; ESAs: erythropoiesis-stimulating agents; MD: mean differences; 95% CI: 95% confidence interval.

**Supplementary Table S4. Subgroup SUCRAs of Hb response.**

| **Model** | **Subgroup** | **SUCRA** | | | | | |
| --- | --- | --- | --- | --- | --- | --- | --- |
|  |  | Daprodustat | Desidustat | ESAs | Molidustat | Roxadustat | Vadadustat |
| Original model | - | 0.44 | 0.98 | 0.55 | 0.08 | 0.78 | 0.16 |
| Subgroup: Mean age | <60 | 0.48 | 0.97 | 0.52 | 0.11 | 0.77 | 0.16 |
|  | ≥60 | 0.46 | 0.96 | 0.57 | 0.10 | 0.75 | 0.16 |
| Subgroup: Male ratio | <0.6 | 0.47 | 0.97 | 0.53 | 0.01 | 0.77 | 0.16 |
|  | ≥0.6 | 0.45 | 0.97 | 0.57 | 0.01 | 0.75 | 0.16 |
| Subgroup: Duration of treatment | <52 weeks | 0.43 | 0.97 | 0.57 | 0.09 | 0.76 | 0.17 |
|  | 52 weeks | 0.44 | 0.97 | 0.55 | 0.09 | 0.78 | 0.17 |

ESAs: erythropoiesis-stimulating agents.

**Supplementary Figure S1. Structure of network formed by treatments.**


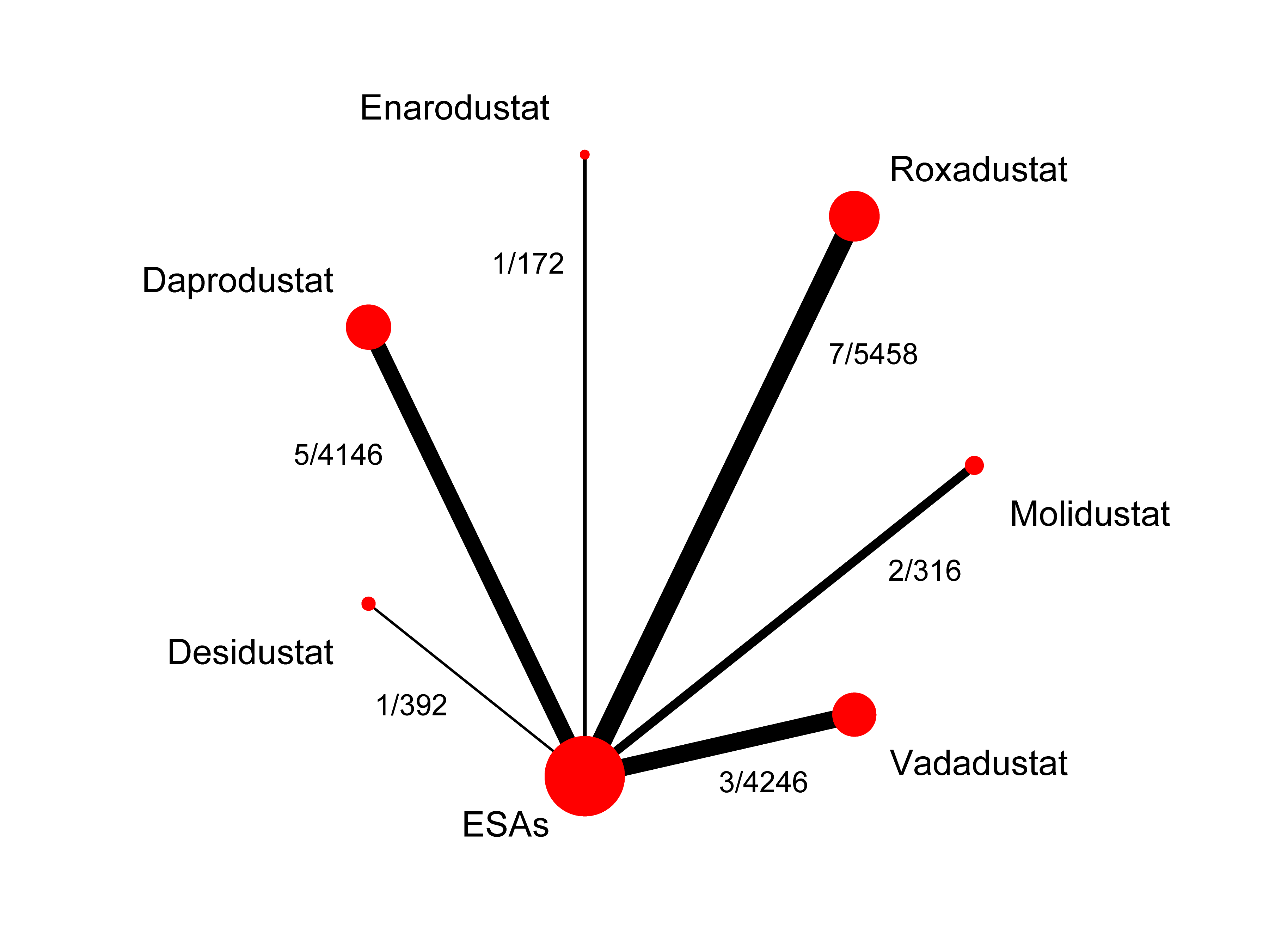


The lines between treatment nodes indicate the direct comparisons made within randomized controlled trials. The width of the lines is proportional to the inverse of standard error, and the sizes of the circles are proportional to the number of patients. Numbers (n/n) near the line indicate “numbers of trials/numbers of participants” of the related comparisons. ESAs: erythropoiesis-stimulating agents.

**Supplementary Figure S2. Risk of bias domains of any adverse event summary of included trials.**


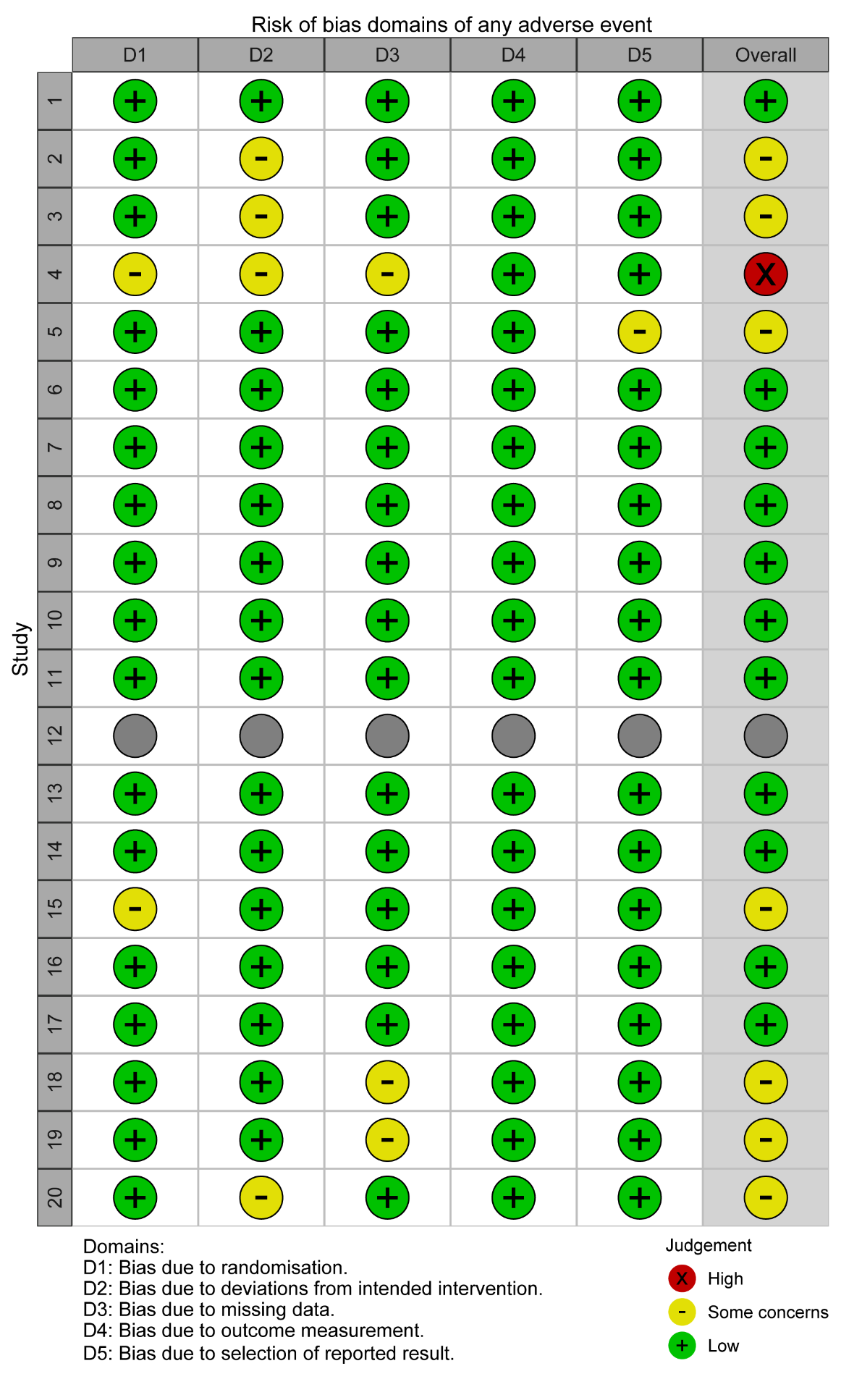


**Supplementary Figure S3. Risk of bias domains of any adverse event summary of included trials.**


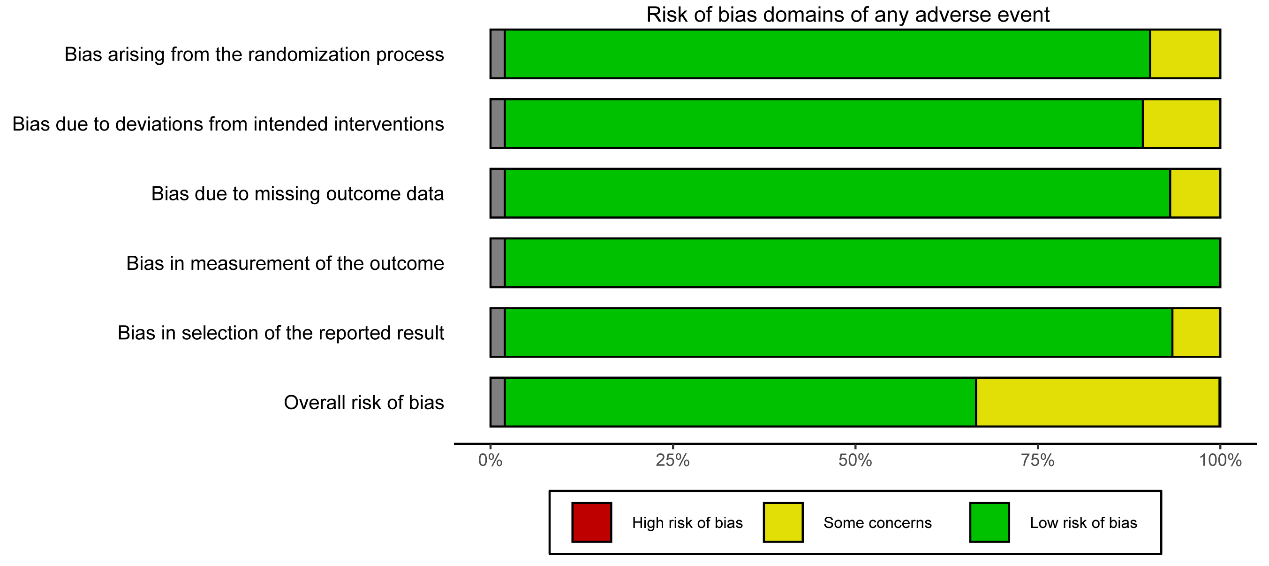


**Supplementary Figure S4. Risk of bias domains of any serious adverse event summary of included trials.**


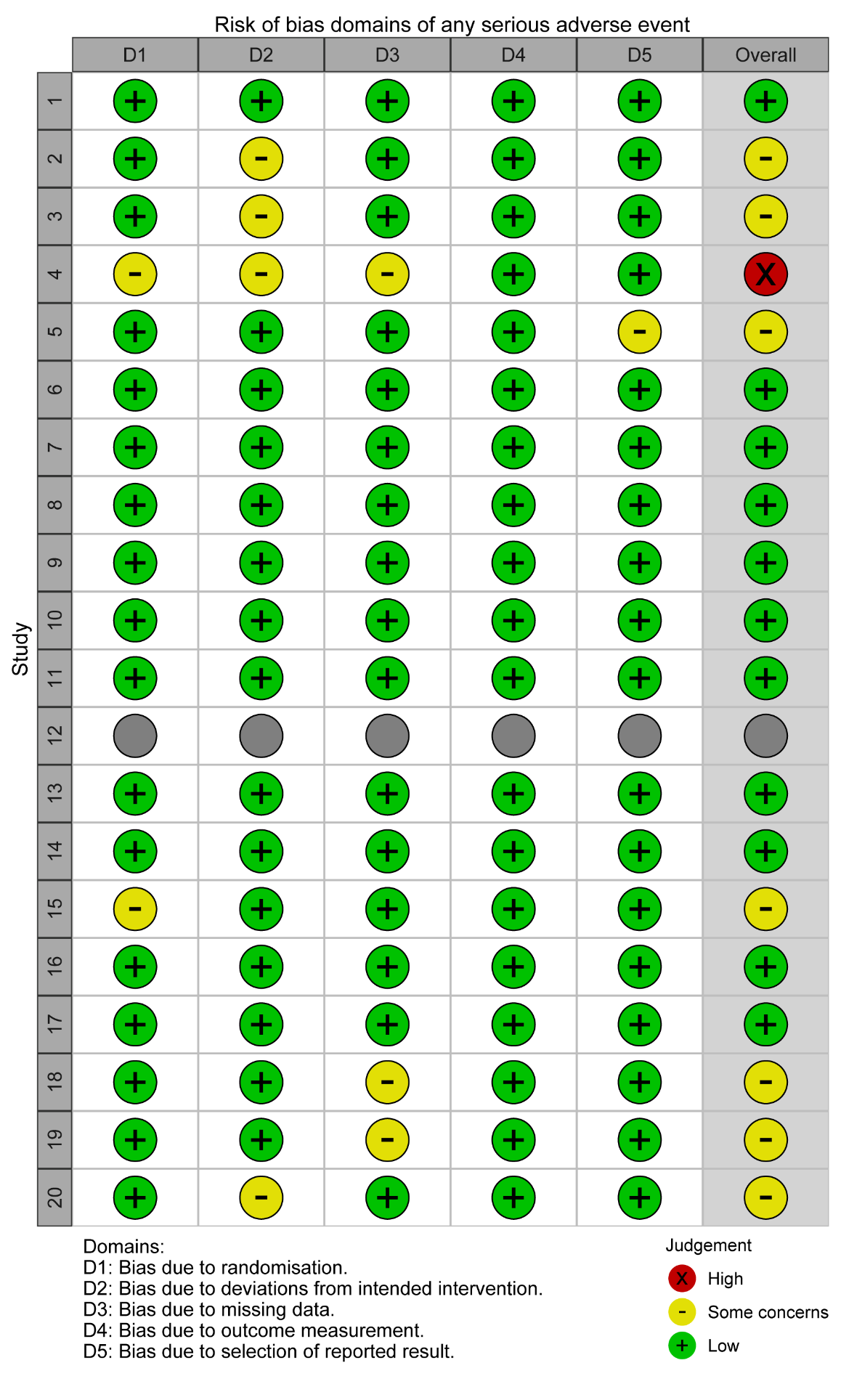


**Supplementary Figure S5. Risk of bias domains of any serious adverse event summary of included trials.**


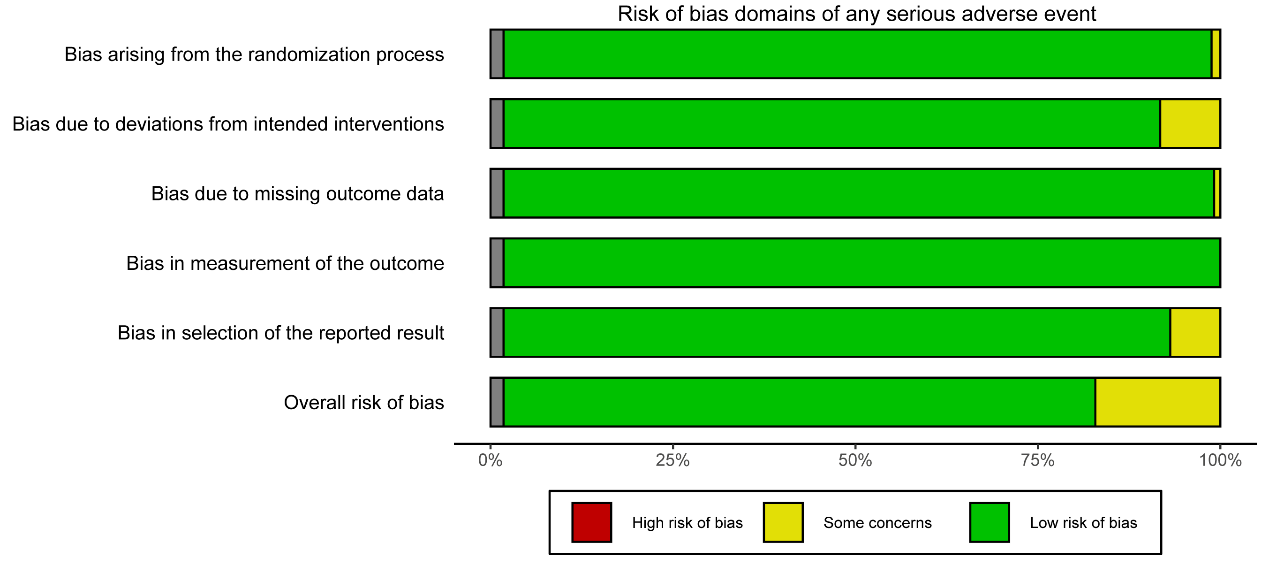


**Supplementary Figure S6. Risk of bias domains of Hb response summary of included trials for DD CKD patients.**


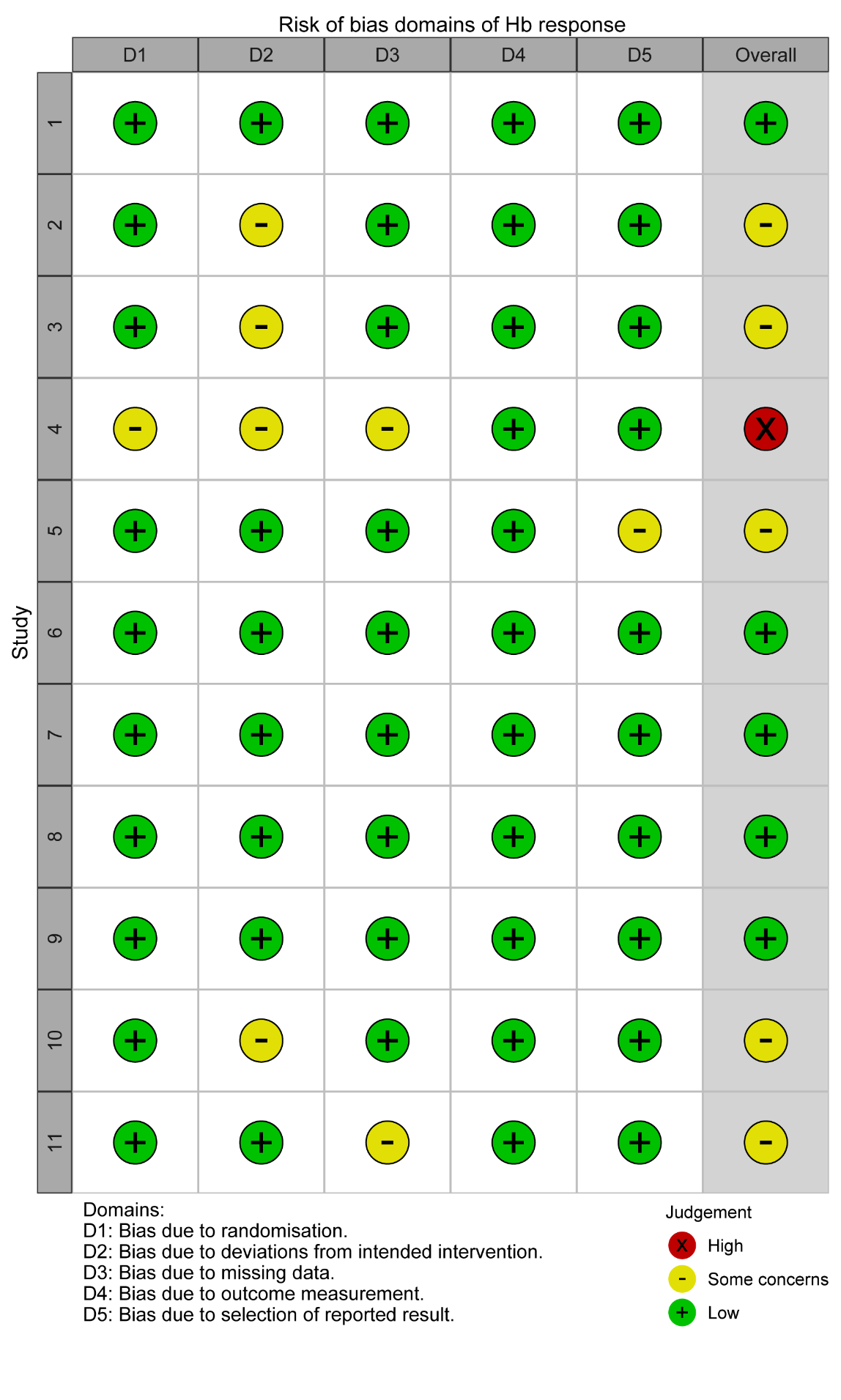


**Supplementary Figure S7. Risk of bias domains of Hb summary of included trials.**


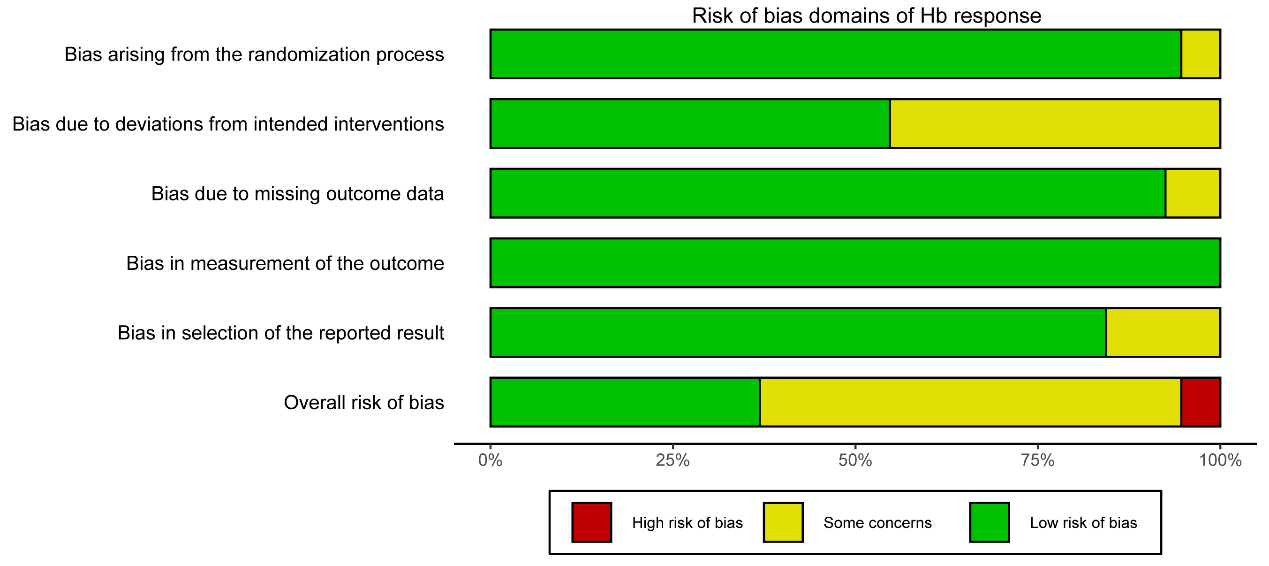


**Supplementary Figure S8. Risk of bias domains of ΔHb summary of included trials.**


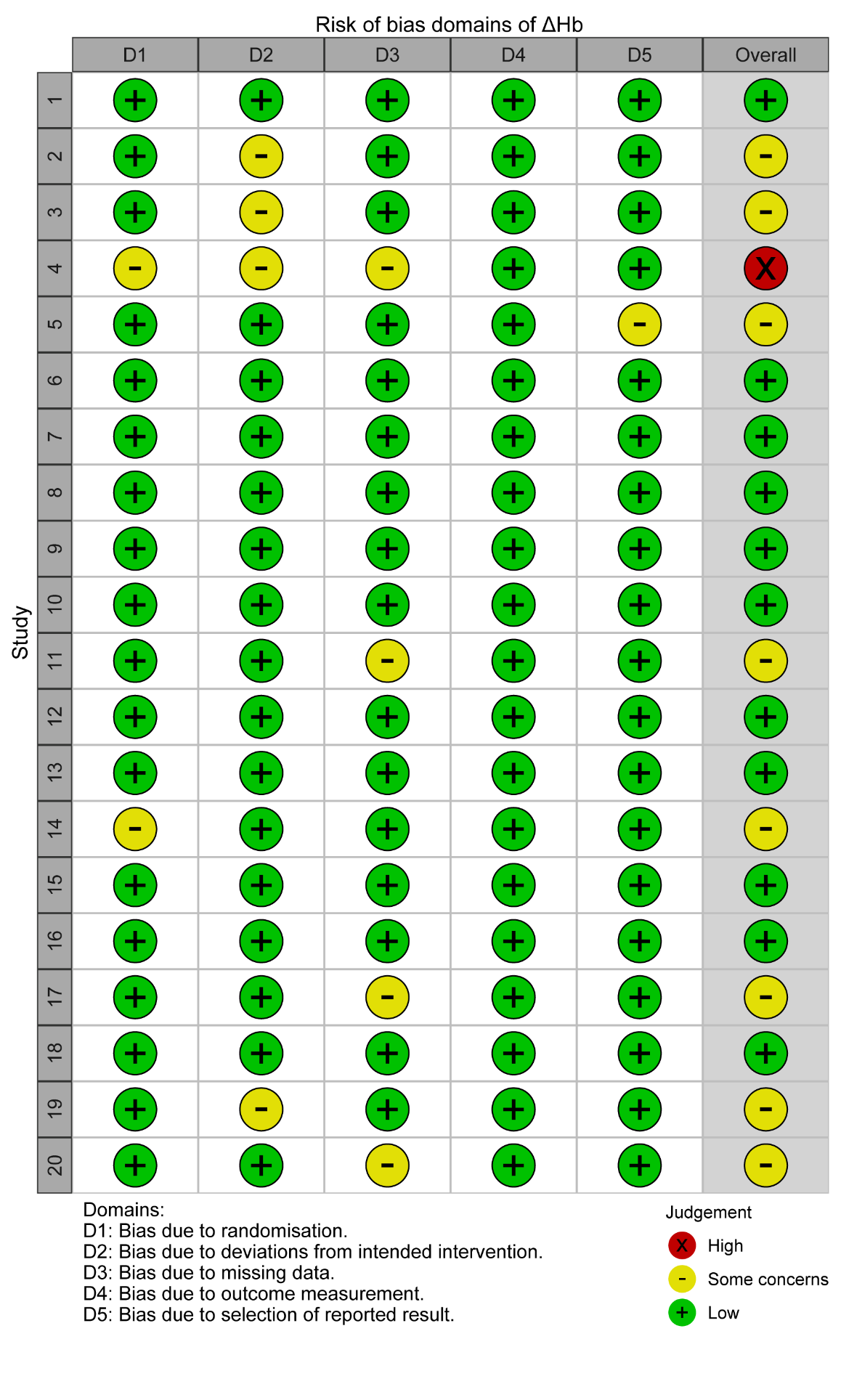


**Supplementary Figure S9. Risk of bias domains of ΔHb summary of included trials.**


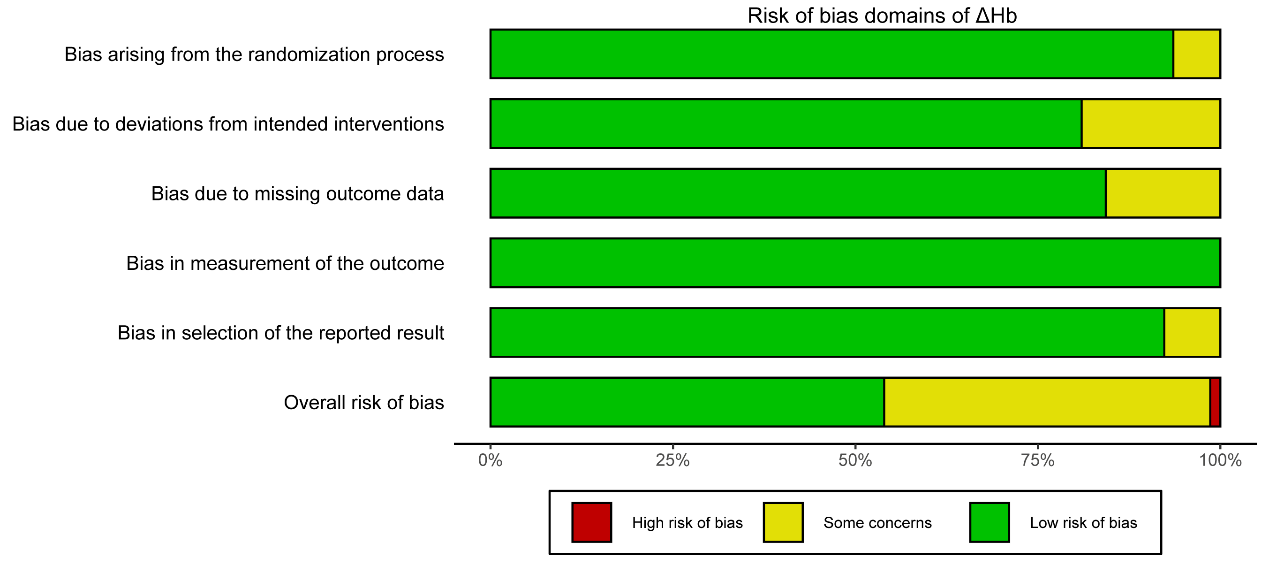


**Supplementary Figure S10. Net-ranking for the safety of any adverse event.**

**
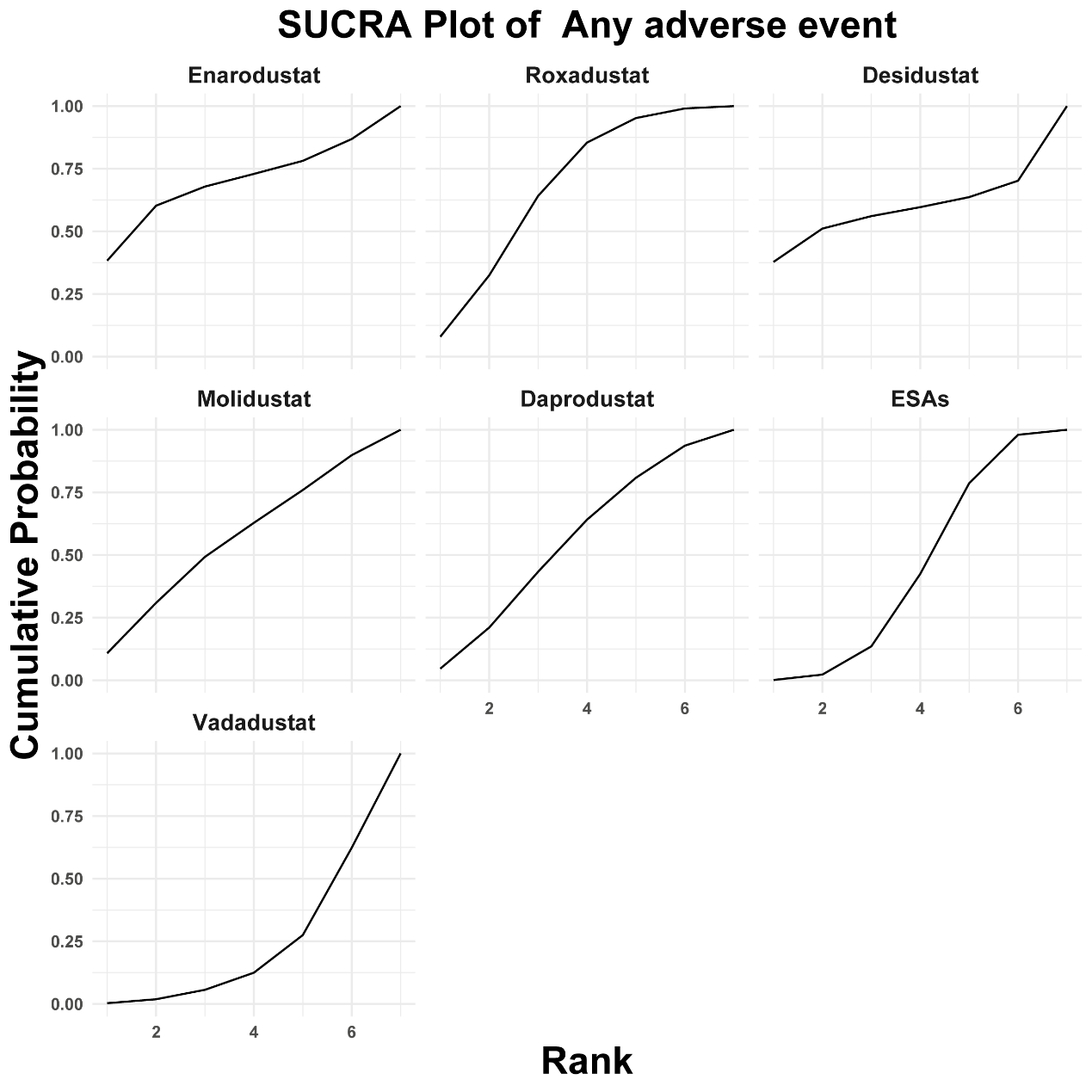
**

P-Score represents the net-ranking of SUCRA score, and its higher score means a higher possibility of any adverse event. ESAs: erythropoiesis-stimulating agents.

**Supplementary Figure S11. Net-ranking for the safety of any serious adverse event.**

**
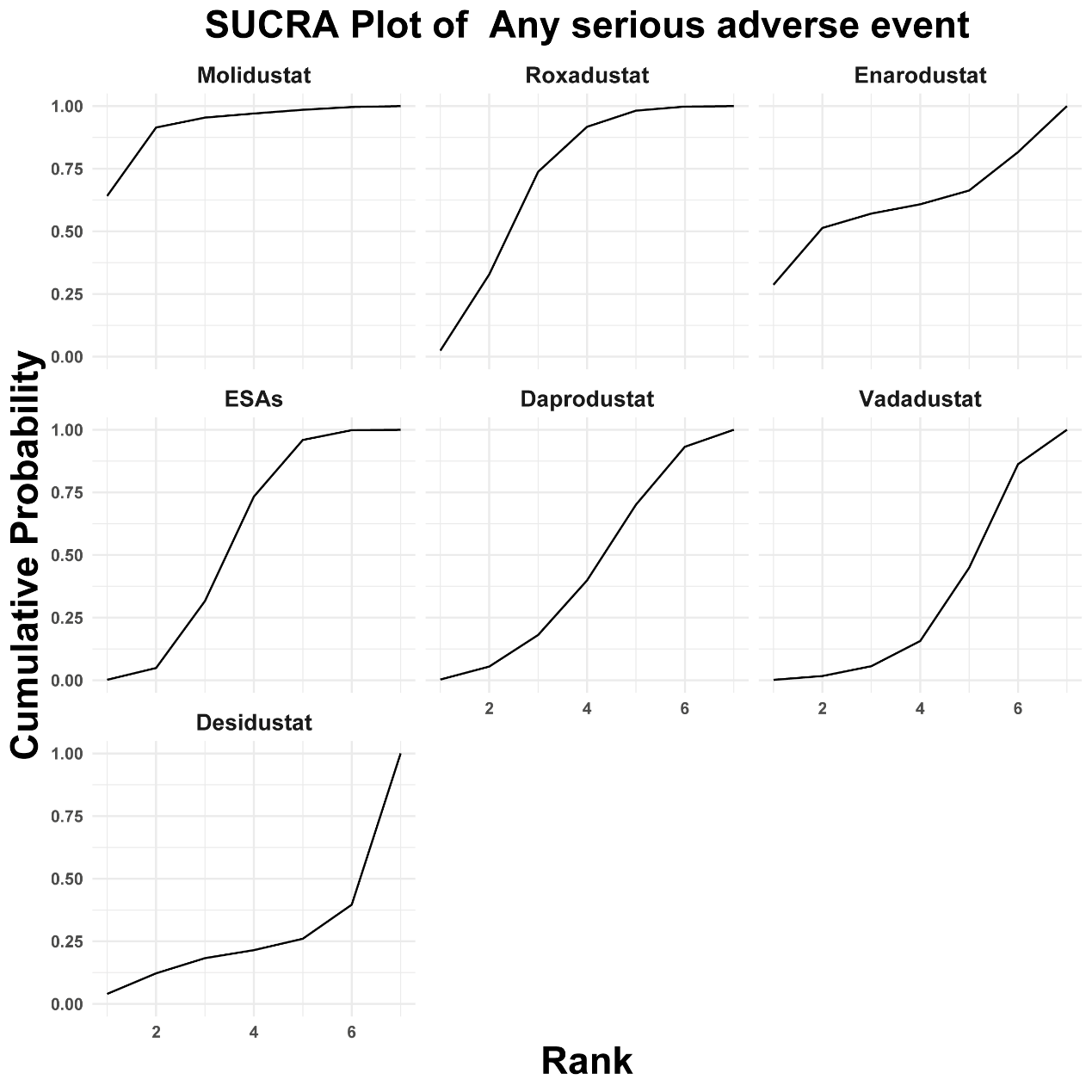
**

P-Score represents the net-ranking of SUCRA score, and its higher score means a higher possibility of any serious adverse event. ESAs: erythropoiesis-stimulating agents.

**Supplementary Figure S12. Net-ranking for Hb response.**

**
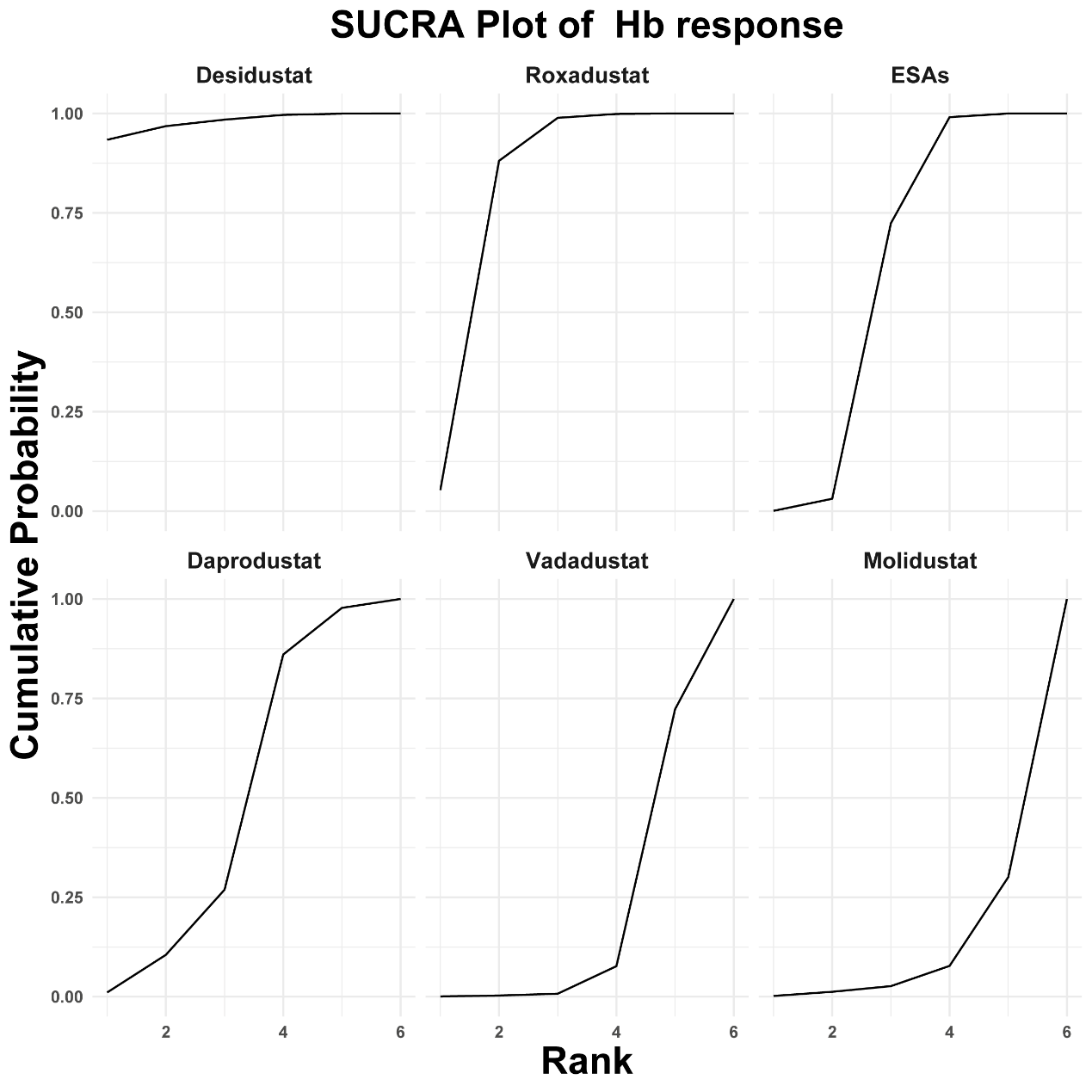
**

P-Score represents the net-ranking of SUCRA score, and its higher score means better performance. ESAs: erythropoiesis-stimulating agents.

**Supplementary Figure S13. Forest plots for the ΔHb.**


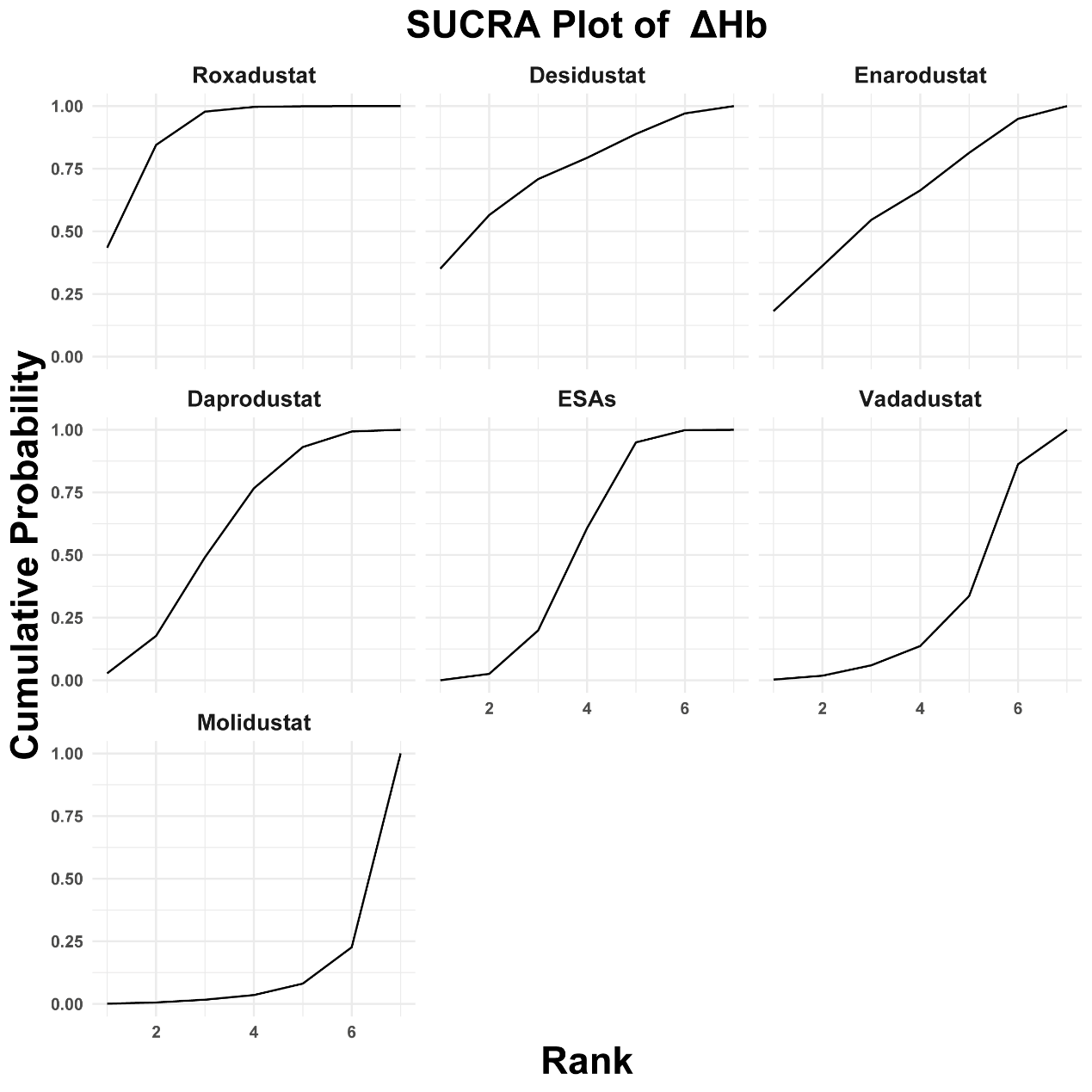


P-Score represents the net-ranking of SUCRA score, and its higher score means better performance. ESAs: erythropoiesis-stimulating agents.

**Supplementary Figure S14. Forest plots for the influence on any adverse event of mean age, male ratio, and duration of treatment.**

**
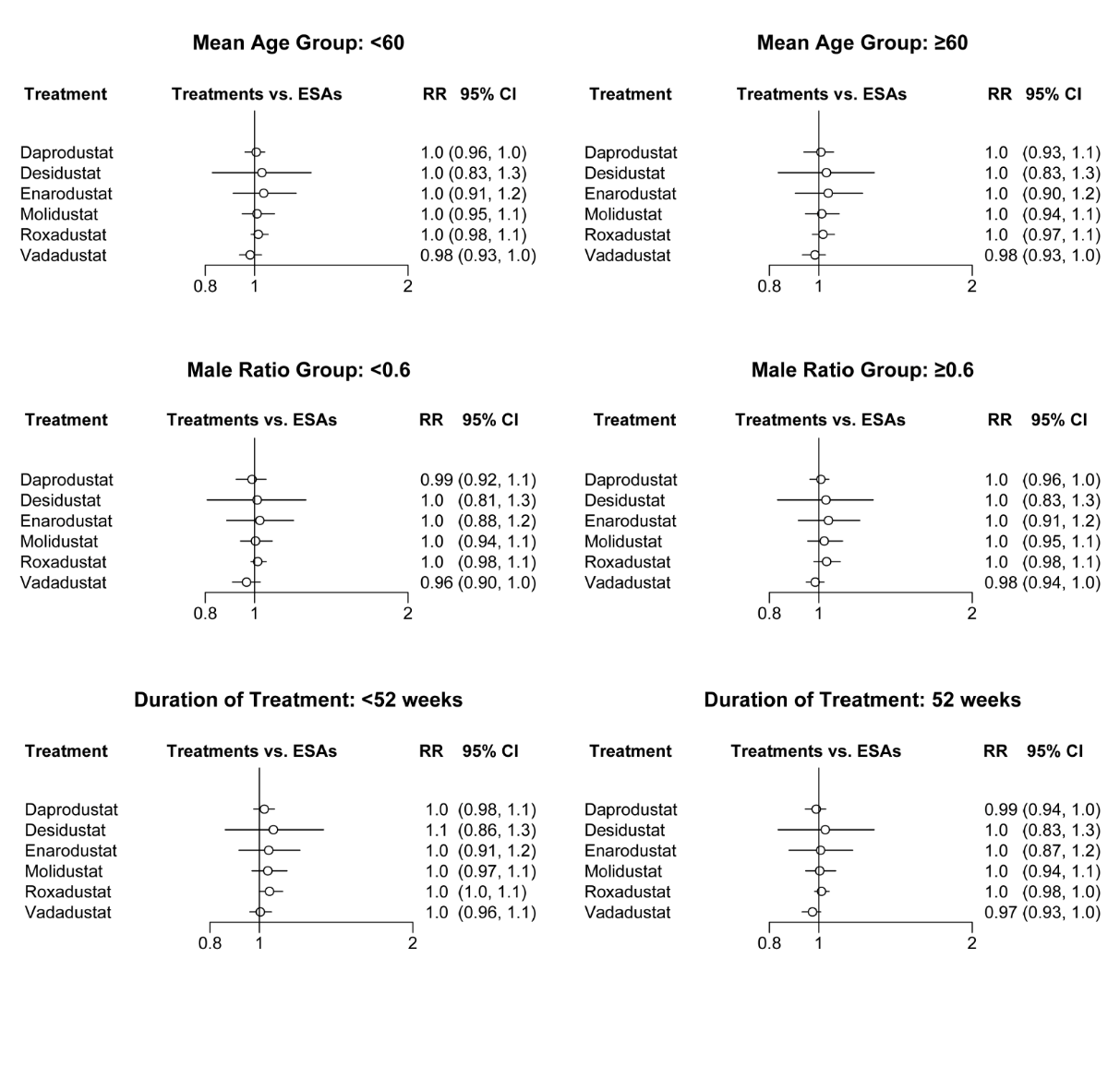
**

ESAs: erythropoiesis-stimulating agents; RR: risk ratio; 95%CI: 95% confidence interval.

**Supplementary Figure S15. Forest plots for the influence on any serious adverse event of mean age, male ratio, and duration of treatment.**

**
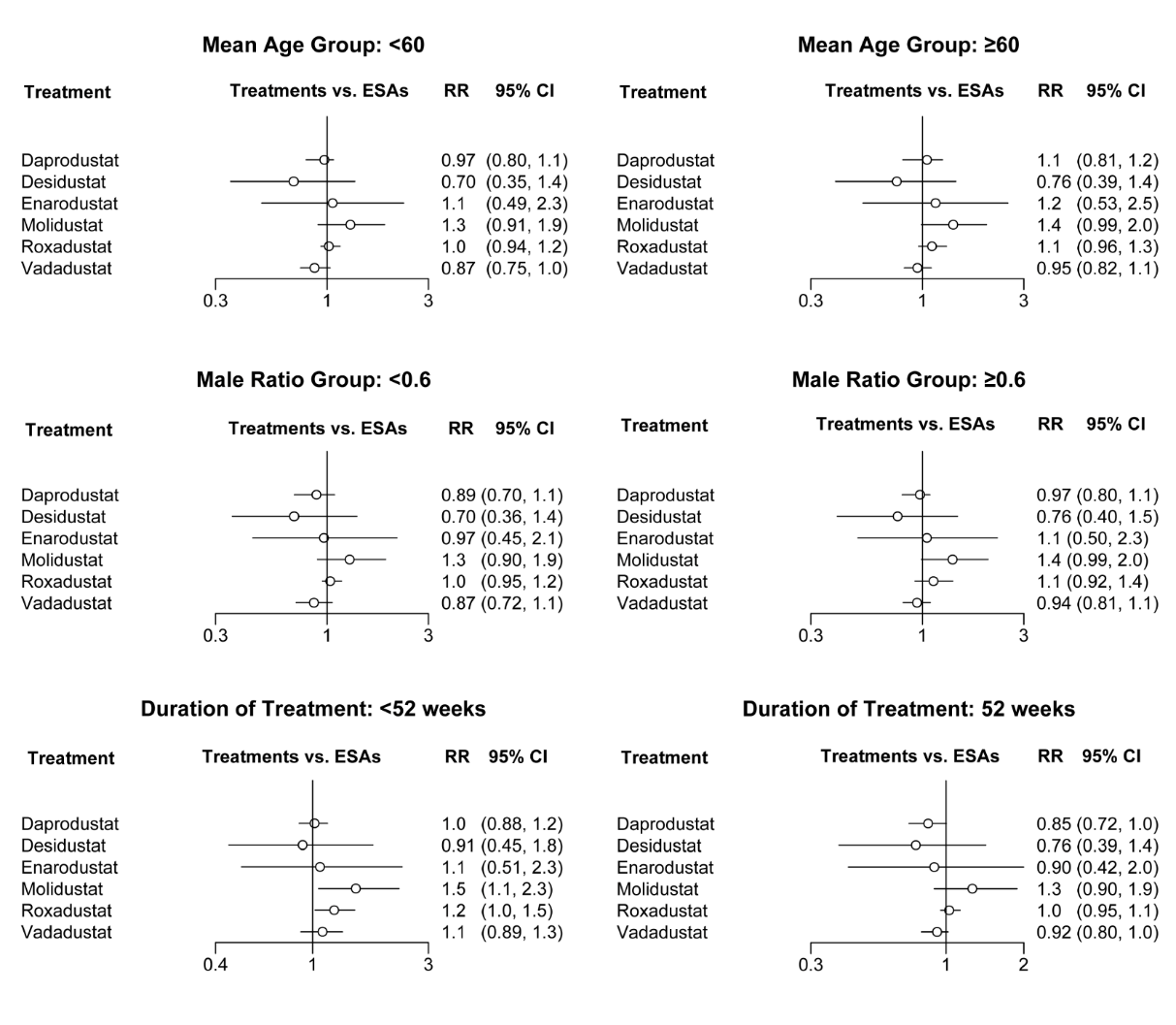
**

ESAs: erythropoiesis-stimulating agents; RR: risk ratio; 95%CI: 95% confidence interval.

**Supplementary Figure S16.** **Forest plots for the ΔHb.**


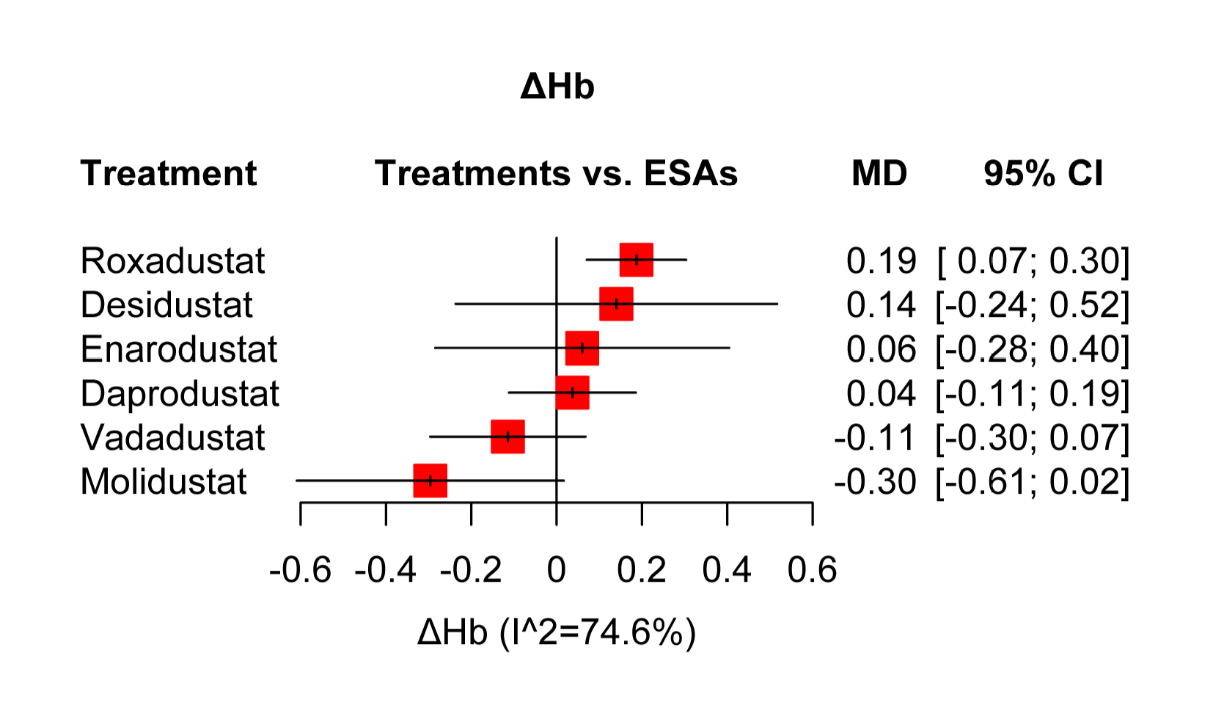


ΔHb: hemoglobin level changed; ESAs: erythropoiesis-stimulating agents; MD: mean differences; 95%CI: 95%confidence interval.

**Supplementary Figure S17. Forest plots for the influence on Hb response of mean age, male ratio, and duration of treatment.**

**
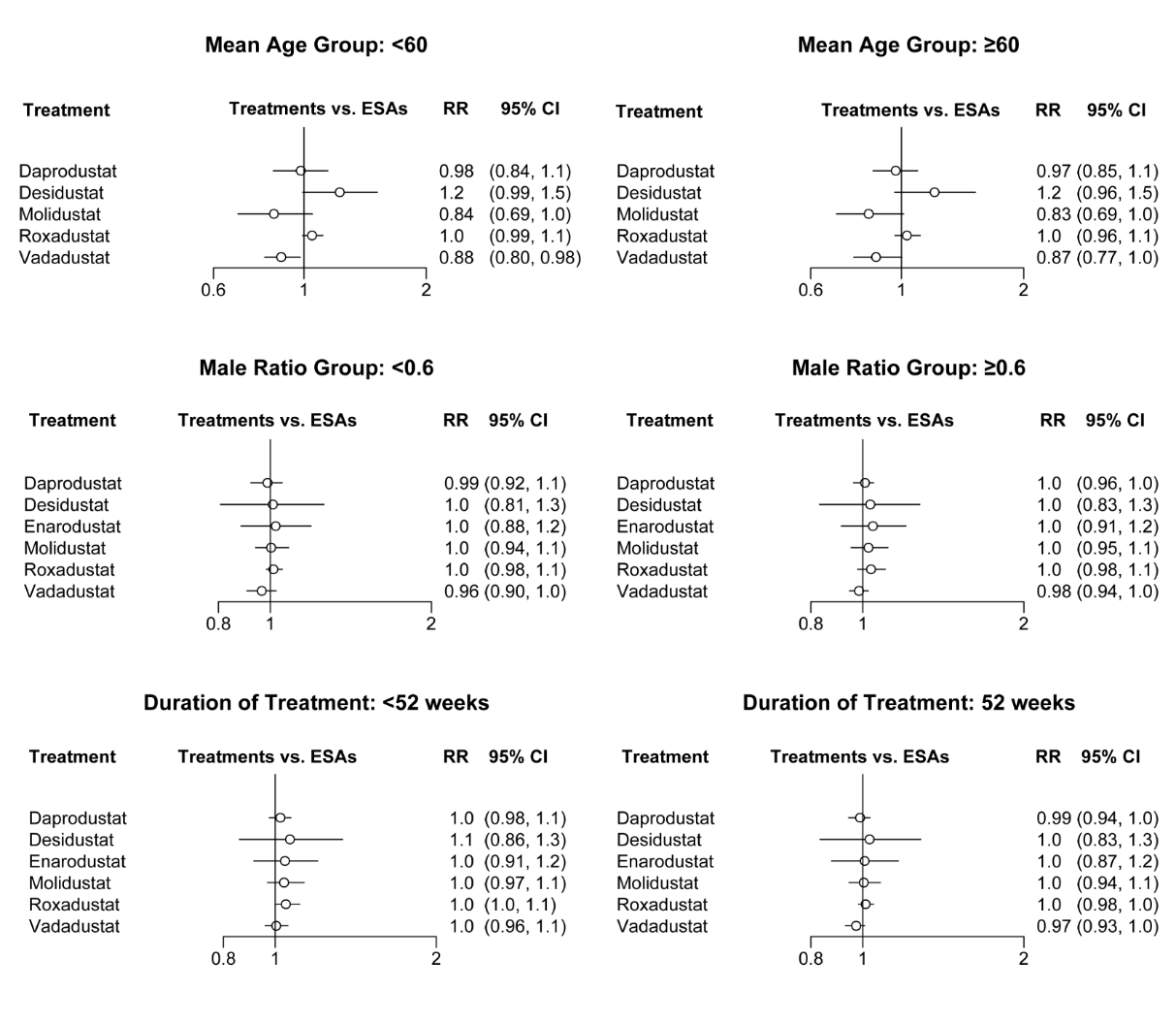
**

ESAs: erythropoiesis-stimulating agents; RR: risk ratio; 95%CI: 95% confidence interval.

**Supplementary Figure S18. Forest plots for the iron metabolism indicators.**

**
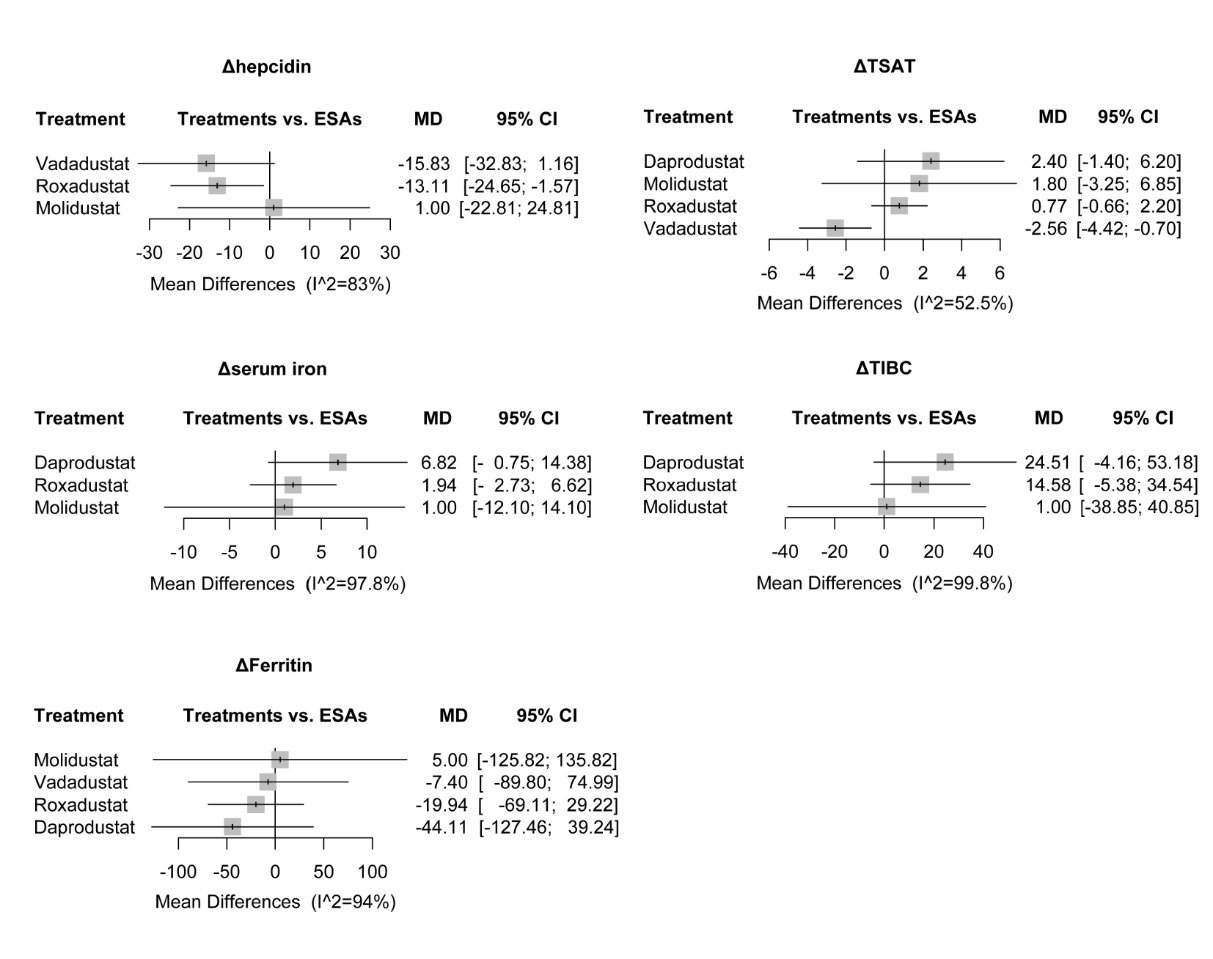
**

ESAs: erythropoiesis-stimulating agents; MD: mean differences; 95% CI: 95% confidence interval.

**Supplementary Figure S19. Comparison-adjusted funnel plots for any adverse event.**


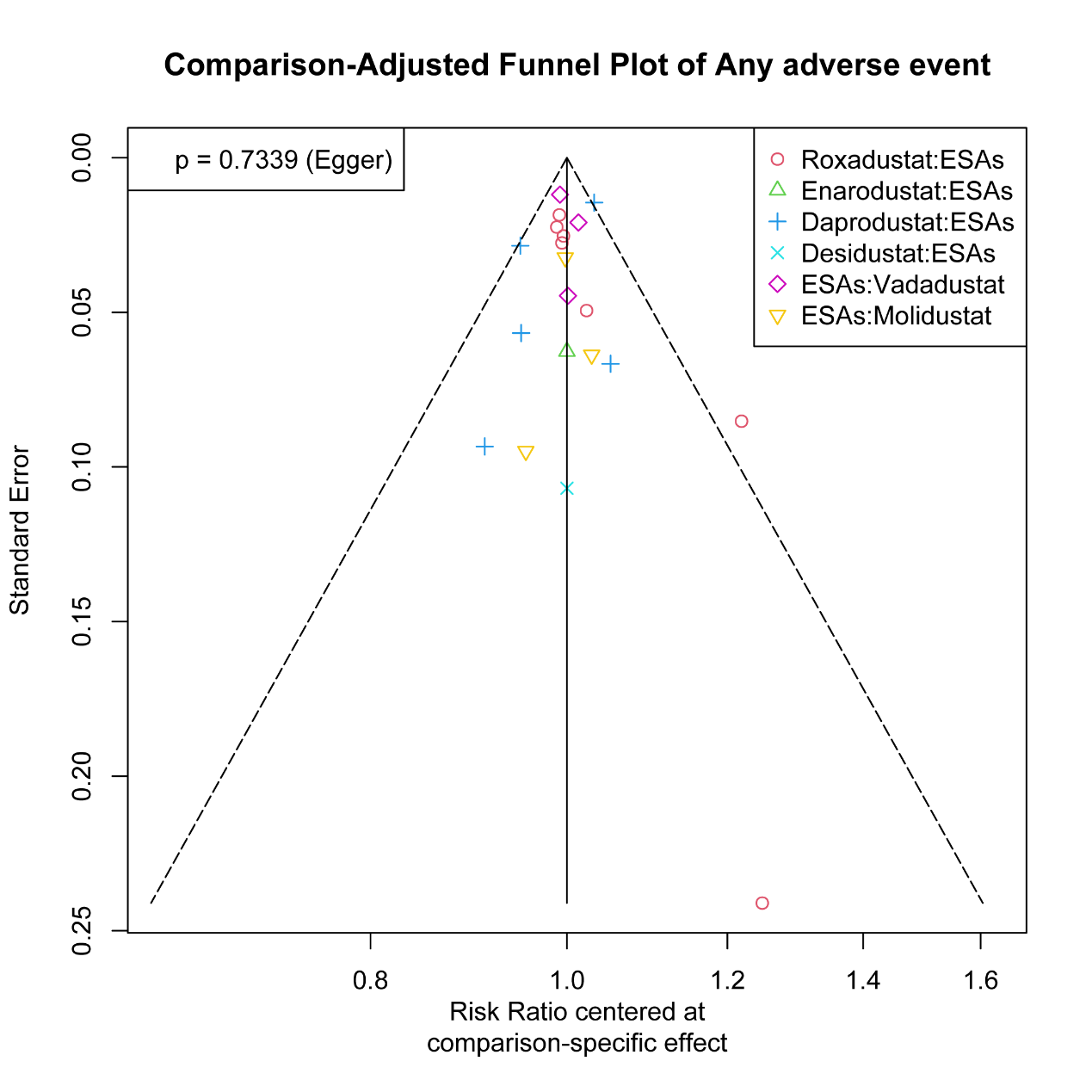


Egger's test; P>0.05. ESAs: erythropoiesis-stimulating agents.

**Supplementary Figure S20. Comparison-adjusted funnel plots for any serious adverse event.**


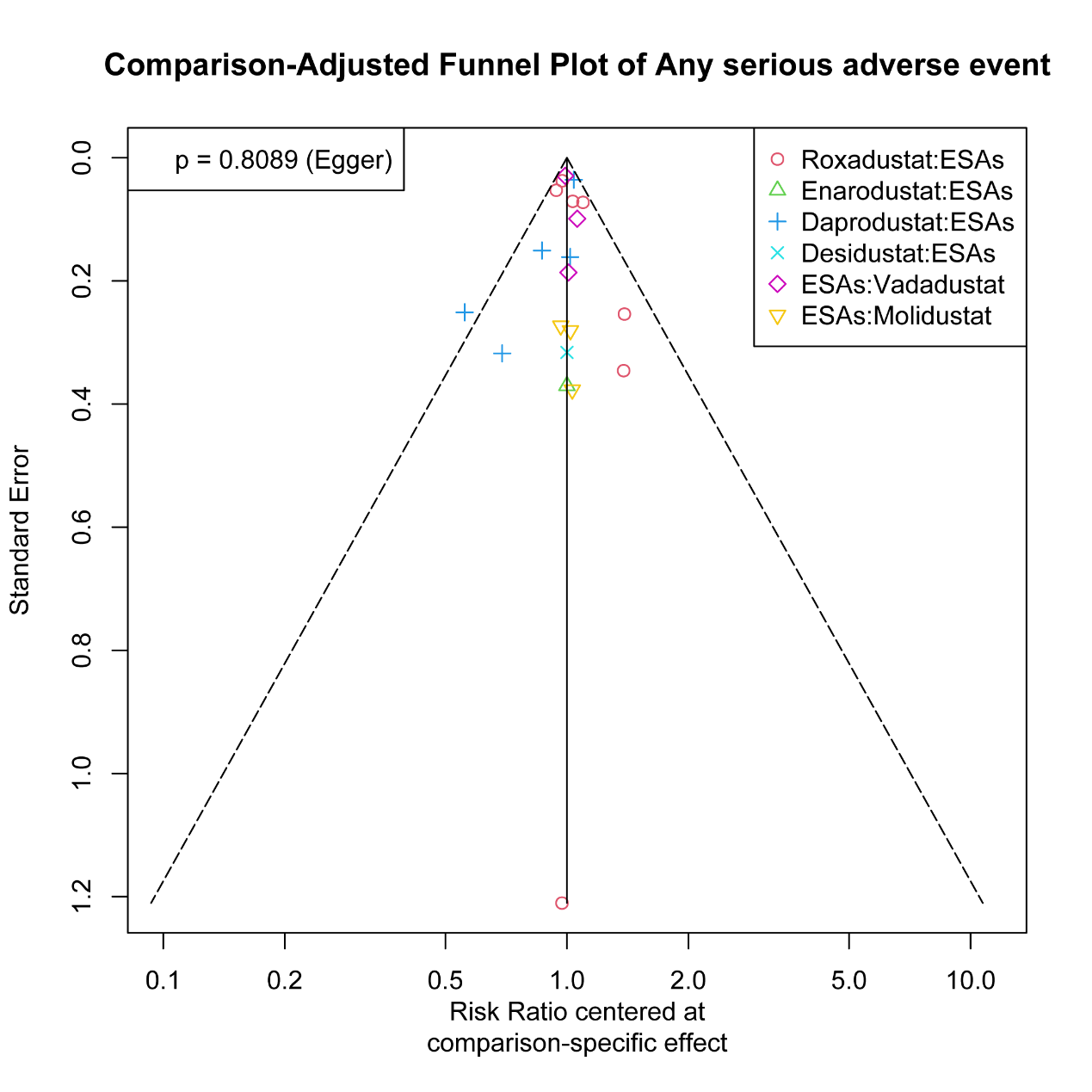


Egger's test; P>0.05. ESAs: erythropoiesis-stimulating agents.

**Supplementary Figure S21. Comparison-adjusted funnel plots for the Hb response.**


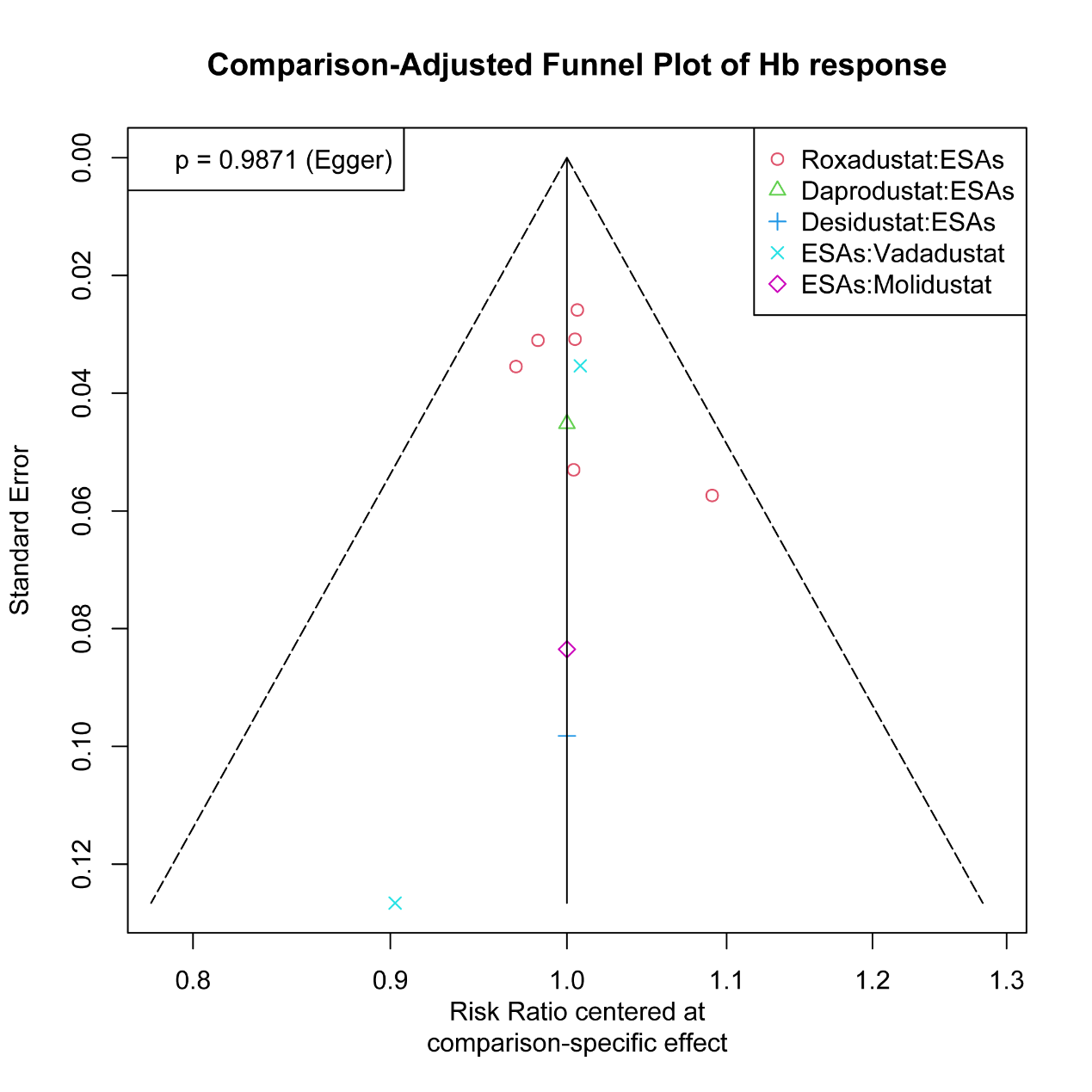


Egger's test; P>0.05. ESAs: erythropoiesis-stimulating agents.

**Supplementary Figure S22. Comparison-adjusted funnel plots for the ΔHb.**


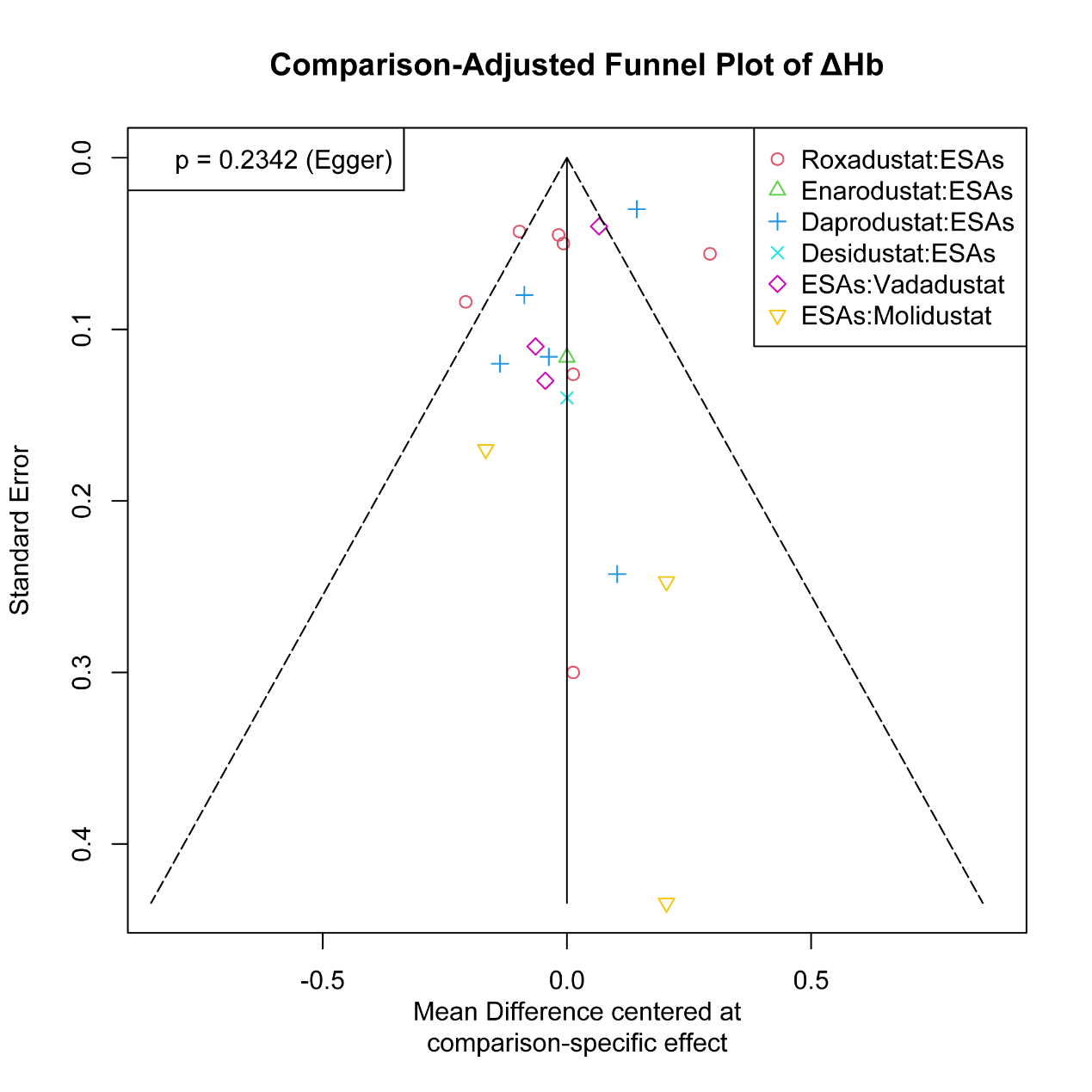


Egger's test; P>0.05. ESAs: erythropoiesis-stimulating agents.
